# Maternal probiotic supplementation and offspring health: an umbrella review with re-analysis of systematic reviews and meta-analyses

**DOI:** 10.3389/fnut.2026.1764109

**Published:** 2026-03-26

**Authors:** Wenrui Sun, Yu Cao, Shumin Li, Liping Sun, Yuexia Liao

**Affiliations:** School of Nursing, Faculty of Medicine, Yangzhou University, Yangzhou, China

**Keywords:** infant, lactation, newborn, pregnancy, probiotics, umbrella review

## Abstract

**Background:**

The maternal gut microbiota critically influences offspring colonization and long-term health. Probiotics have attracted significant attention due to their microbiota-modulating potential. However, the lack of clear international guidelines for their use in pregnancy and lactation underscores the need for robust evidence on safety and the impact of maternal supplementation on offspring health.

**Objective:**

We conducted an umbrella review (UR) of systematic reviews (SRs) with meta-analyses (MAs) to evaluate the quality, certainty, and credibility of evidence regarding the effects of maternal probiotic supplementation during pregnancy and/or lactation on offspring health.

**Methods:**

We searched PubMed, Embase, CINAHL, Web of Science, and Cochrane Library up to June 15, 2025, for peer-reviewed SRs. Summary effects were re-estimated using random-effects models. Quantitative evidence quality was appraised with AMSTAR-2 and certainty graded using GRADE. Outcomes from single primary studies or with unextractable data were summarized narratively.

**Results:**

Eighteen SRs were included, of which 13 provided MAs with ≥2 original studies, encompassing 62 specific offspring-related outcomes. Among these, three types of outcomes were rated as having moderate to high certainty by GRADE. Maternal probiotic supplementation was associated with lower risks of eczema or atopic eczema in offspring, primarily within the first 1–2 years of life (e.g., RR = 0.52, 95% CI: 0.38 to 0.72; *p* < 0.001), and decreased abundance of pathogenic bacteria in breast milk (e.g., SMD = −0.90, 95% CI: −1.49 to −0.31; *p* = 0.003). Nevertheless, according to the UR credibility criteria, all statistically significant associations were rated as having weak credibility. Sensitivity analyses using more conservative methods showed that 65% of these associations lost significance, though the associations with eczema and breast milk microbiota improvement remained significant.

**Conclusion:**

Current evidence remains insufficient to confirm benefits or harms of maternal probiotic supplementation. Although statistical associations were observed for eczema risk and pathogenic bacteria in breast milk, weak credibility of these findings necessitates further robust studies to verify these effects.

**Systematic review registration:**

https://www.crd.york.ac.uk/PROSPERO/view/CRD420251120972, PROSPERO CRD420251120972.

## 1 Introduction

In recent decades, the gut microbiota has gained increasing recognition for its critical role in immunity (1), metabolism (2), and other physiological processes. This understanding has expanded to include the maternal microbiota, whose modulation has been identified as a key factor in offspring gut colonization (3) and long-term health (4). While the underlying mechanisms remain poorly understood, this uncertainty has spurred a growing body of research into probiotics—live microorganisms which, when consumed in adequate amounts through food or supplements, confer health benefits to the host (5). Research on probiotics has advanced significantly, demonstrating their broad therapeutic potential, from modulating the gut microbiota (6) to supporting immune health (7).

In recent years, the increasing awareness of gut health and preventive healthcare has driven the rapid expansion of the probiotic industry, with the global market size reaching 87.7 billion US dollars in 2023 (8). This trend is especially pronounced among younger individuals, particularly women, with pregnant and lactating women forming a distinct and rapidly growing consumer subgroup (9). However, this surge in popularity contrasts sharply with significant evidence gaps. To date, the U.S. Food and Drug Administration has not approved any probiotic product as a drug or biological product for infants (0–12 months) due to insufficient evidence regarding their efficacy and safety (10). Moreover, to our knowledge, dietary guidelines from the UpToDate database, as well as society- or government-sponsored probiotic frameworks worldwide, provide no specific recommendations on probiotic use during pregnancy or lactation (11, 12). This discrepancy between widespread use and limited evidence raises concerns about potential risks to maternal pregnancy outcomes and infant health, including rare probiotic-associated infections in vulnerable individuals and largely theoretical effects on neonates through maternal supplementation (13).

Therefore, we conducted an umbrella review (UR) to systematically evaluate the safety and efficacy of maternal probiotic supplementation, with a specific focus on its potential impact on offspring health outcomes through quantitative and narrative synthesis. Our study aims to fill critical evidence gaps and provide insights that can inform both clinical practice and the commercial development of maternal probiotic supplementation.

## 2 Methods

### 2.1 Literature search

In the UR of meta-analyses (MAs) and qualitative synthesis, we systematically searched PubMed (MEDLINE), Embase, CINAHL, Web of Science, and Cochrane Library from their inception to June 15, 2025. The comprehensive search strategy using Boolean logic and relevant keywords and MeSH terms is available in Supplementary file 1. Then we identified peer-reviewed systematic reviews (SRs), with and without Mas, that evaluated the associations between maternal probiotic supplementation during pregnancy and/or lactation and offspring health-related outcomes and assessed the direction of these effects. We included English-language articles without restrictions on primary study design. This study is registered with PROSPERO (CRD420251120972).^1^

### 2.2 Eligibility criteria

We included articles if they met the following PICOS (Population, Intervention, Comparison, Outcome, and Study design) criteria: (1) Population: to avoid confounding factors specific to adolescent pregnancy, we included pregnant and/or lactating women aged at least 18 years at any gestational stage, without conditions impairing probiotic intake or absorption; (2) Intervention: probiotic supplementation administered via any route (e.g., oral or vaginal) and in any formulation (e.g., capsule, powder, oil drop, yogurt, or other fermented foods and beverages), with no restrictions on probiotic dosage, species, strains (e.g., *Lactobacillus rhamnosus* GG, *Bifidobacterium lactis* BB-12), or strain composition (single- or multi-strain), and administered for at least one consecutive week during pregnancy or lactation; (3) Comparison: placebo, standard treatment, or probiotics added to a therapy versus the same therapy with placebo; (4) Outcome: infant diseases (e.g., eczema or sepsis), birth outcomes (e.g., preterm birth or low birth weight), delivery modes (e.g., vaginal delivery or cesarean section), and relevant biomarkers; and (5) Study design: SRs with or without MAs, without restrictions on the design of the primary studies.

Exclusion criteria: (1) Animal studies; (2) Articles not published in English; (3) Non-peer-reviewed publications; (4) Studies including prebiotics or synbiotics where the specific effect of the probiotic could not be isolated; (5) Broad dietary interventions lacking targeted probiotic supplementation; (6) Studies involving the direct administration of probiotics to infants.

Two investigators (S-WR and C-Y) independently screened titles and abstracts of identified records and selected articles after full-text review. Any discrepancies were resolved through discussion.

### 2.3 Data extraction

Data on key indicators (e.g., first author, journal, publication year, outcomes of interest, and the number of studies included) were extracted from each eligible MA by one investigator and verified by the other (Supplementary file 2). These indicators were recorded at the MA level and used to identify and link all corresponding outcome-level associations derived from each MA. Each comparison of an offspring outcome associated with maternal probiotic exposure was treated as a unique MA, regardless of whether it was reported as a primary or secondary outcome (14, 15). Subgroup analyses in included MAs were considered separately, and only independent datasets were retained. To minimize bias from duplicated evidence, overlap among MAs was assessed systematically using the corrected covered area (CCA), with duplicated associations excluded as appropriate (16). Additionally, MAs evaluating multiple outcomes of interest were recorded separately (17).

Extracted outcome-level associations were stratified by supplementation timing and categorized according to outcome domains. For MAs assessing similar associations within a domain, a pairwise overlap matrix was developed to calculate the CCA between each pair (16, 18). Overlap was classified as slight (0–5%), moderate (6–10%), high (11–15%), or very high (>15%) (18). When high or very high overlap for the same outcome-level association was identified across meta-analyses, a single association was retained for quantitative synthesis to avoid double-counting of primary studies. Selection followed a pre-specified hierarchical rule, prioritizing the association from the MA with higher AMSTAR 2 ratings (19), followed by more recent publication year, and then broader population coverage or larger numbers of included trials. When all extractable associations from a single MA were superseded by similar associations from other MAs, the entire MA was excluded from quantitative synthesis.

We extracted means, standard deviations, and group sizes for continuous outcomes, and event counts and group sizes for dichotomous outcomes, as reported in the MAs (Supplementary file 3). When relevant information was not fully available in the MAs, we traced back to the corresponding primary studies to obtain the necessary data. These data were used in the subsequent quantitative analyses.

### 2.4 Statistical analysis

Summary effect sizes and 95% confidence intervals (CI) for all eligible MAs were re-estimated using the random-effects DerSimonian and Laird (DL) model to ensure methodological consistency across the studies (20).

In MAs with continuous data, results were interpreted as equivalent standardized mean differences (SMD), where the direction (positive or negative) indicates benefit or risk, depending on the specific offspring outcomes. For MAs with dichotomous data, results were expressed as risk ratios (RR), and the interpretation of RR relative to 1 varies based on the specific outcome.

Between-study heterogeneity was assessed using the *I*^2^ statistic, with values greater than 50 and 75% representing large and very large heterogeneity, respectively (21).

We also calculated a 95% prediction interval (PI) for each outcome to estimate the potential range of effects that a new study examining the same association might observe, considering the uncertainty in the overall effect and heterogeneity (22). These intervals provide a more conservative estimate than the 95% CI.

Egger’s regression asymmetry test (*p* ≤ 0.10) was applied to assess potential small-study effects, specifically whether smaller studies tend to overestimate the effect size in each MA (23, 24). Additionally, we evaluated the small-study effects by considering whether the largest study (i.e., the study with the smallest standard error), which had a more conservative effect estimate than the random effects MA, was statistically significant (25).

Finally, the excess statistical significance test was used to evaluate whether the observed number of studies (*O*) with nominally significant results (*p* < 0.05) exceeded the expected number (*E*) (26). This test investigates whether the number of positive studies in a MA is disproportionately high, given their statistical power to detect plausible effects at *α* = 0.05. We calculated the statistical power of each study in the MA and determined *E* by summing these values (27). The power of each study was estimated based on the effect size of the largest study in the MA, using an algorithm with a non-central *t* distribution (28). Excess statistical significance in each MA was indicated by *p* < 0.10 (one-sided) with *O* > *E* (26).

All analyses were conducted using STATA 18 (StataCorp), CMA 3 (Biostat Inc), and SPSS 27 (IBM).

### 2.5 Assessment of certainty and credibility of evidence

The certainty of evidence in the quantitative synthesis was evaluated using the Grading of Recommendations, Assessment, Development, and Evaluation (GRADE) framework, in accordance with the GRADE Handbook (29, 30). Assessments considered risk of bias, inconsistency, indirectness, imprecision, and publication bias, using GRADEpro^2^ (29). Each outcome-level association was then classified into one of four levels: high, moderate, low, or very low (29). For clarity, the definitions of GRADE certainty levels used for interpretation are summarized in Table 1.

**Table 1:** Definitions of GRADE certainty levels.

Certainty level	Definition
High	We are very confident that the true effect lies close to that of the estimate of the effect.
Moderate	We are moderately confident in the effect estimate. The true effect is likely to be close to the estimate of the effect, but there is a possibility that it is substantially different.
Low	Our confidence in the effect estimate is limited. The true effect may be substantially different from the estimate of the effect.
Very low	We have very little confidence in the effect estimate. The true effect is likely to be substantially different from the estimate of the effect.

GRADE, the grading of recommendations, assessment, development, and evaluation. Certainty level definitions were adapted from the GRADE Handbook (30).

Credibility grading is applied to assess the robustness of nominally significant (*p* < 0.05) MAs for both randomized controlled trials (RCTs) and observational studies in URs (25, 31, 32). This grading considered our re-estimated indicators such as sample size, summary effect size, small-study effects, excess statistical significance, heterogeneity, and 95% PIs (Table 2) (25, 31, 32), categorizing the evidence as convincing (class I), highly suggestive (class II), suggestive (class III), or weak (class IV) (25, 31, 32).

**Table 2:** Credibility criteria of associations.

Classification	Criteria
Convincing (class I)	• >1,000 cases • Associations with *p* < 10^−6^ (summary effect) • No significance of small-study effects (*p* > 0.10) • No evidence of excess significance bias (*p* > 0.10) • Prediction intervals not including the null • Largest study nominally significant (*p* < 0.05) • No large heterogeneity (*I*^2^ < 50%)
Highly suggestive (class II)	• >1,000 cases • Associations with *p* < 10^−6^ (summary effect) • Largest study nominally significant (*p* < 0.05)
Suggestive (class III)	• >1,000 cases • Associations with *p* < 10^−3^ (summary effect)
Weak (class IV)	• Remaining statistically significant associations with *p* < 0.05
No significant (NS)	• All results with *p* > 0.05

All assessments were independently conducted by two reviewers (S-WR and C-Y); discrepancies were resolved by consensus.

### 2.6 Methodological quality of evidence

The methodological quality of our quantitative synthesis was assessed using the AMSTAR 2 (a Measurement Tool to Assess Systematic Reviews, revised) checklist by two independent investigators (S-WR and C-Y) (19).

### 2.7 Narrative synthesis

Outcomes based on a single primary study or with unextractable data were narratively synthesized.

## 3 Results

### 3.1 Literature review

**Figure 1:**
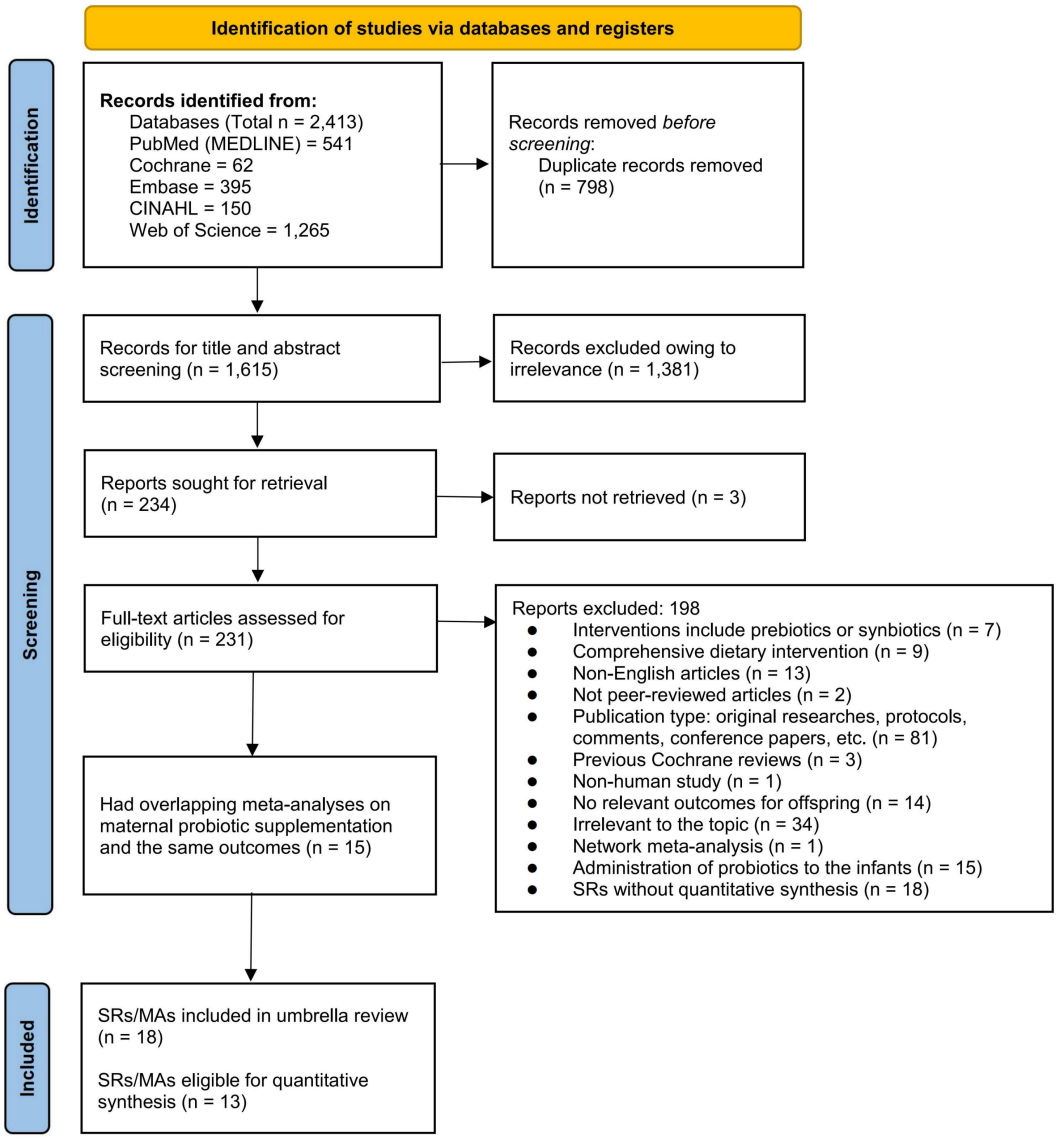
Flow chart of study search and selection.

Overall, we initially identified 1,265 records from the databases. Based on the eligibility criteria, 231 full-text articles were scrutinized, and 18 publications were ultimately included (Figure 1). Of these, 13 (33–45) provided relevant MAs with ≥2 original studies, all of which included RCTs and qualified for inclusion in the quantitative synthesis. Five MAs were excluded from the quantitative synthesis because their relevant outcome-level associations were each based on a single RCT and were synthesized narratively. Fifteen of the 231 full-text articles were excluded based on overlap assessment because all extractable outcome-level associations were fully overlapped by associations retained from other MAs (CCA > 10%). Details and reasons for the exclusion of the remaining 198 reviews are provided in Supplementary file 4.

### 3.2 Quantitative synthesis

#### 3.2.1 Characteristics of systematic reviews and meta-analyses in quantitative synthesis

Thirteen SRs with relevant MAs, corresponding to 64 RCTs, were included in our quantitative synthesis, with their characteristics detailed in Supplementary file 5. The evidence was stratified according to the timing of maternal probiotic supplementation: pregnancy (9 MAs, 36 RCTs), lactation (1 MA, 6 RCTs), both pregnancy and lactation (3 MAs, 32 RCTs), or variable intervention timing (pregnancy and/or lactation; 5 MAs, 18 RCTs) (Table 3).

**Table 3:** Associations with weak credibility (class IV) in meta-analyses between maternal probiotic supplementation and offspring health outcomes.

Outcome	Source	No. of included studies per association	Metric	Effect size	Criteria of credibility grading								
					95% CI	*I^2^* (%)	*p* (heterogeneity)	*p* (overall effect)	No. of events (dichotomous data) Sample size (continuous data)	95% PI	SSE/ESB	LS	Credibility grading
Pregnancy													
Fetal growth and development													
Birth weight	Saeed Baradwan, 2023	4	SMD	0.36	0.12 to 0.61	19.8	0.291	0.003	339	−0.349 to 1.079	Yes/no	No	Weak (IV)
	Bekalu Kassie Alemu, 2023	2	SMD	−0.50	−0.83 to −0.17	0	0.585	0.003	145	NA		Yes	Weak (IV)
Timing of delivery													
Gestational age at birth	Saeed Baradwan, 2023	4	SMD	0.63	0.03 to 1.23	86	0	0.039	339	−2.138 to 3.405	Yes/no	No	Weak (IV)
Breast milk microbiota													
Detection rate of beneficial bacteria in breast milk	Bekalu Kassie Alemu, 2023	6	RR	1.95	1.03 to 3.66	62.7	0.03	0.039	253/654	0.292 to 12.991	Yes/no	Yes	Weak (IV)
Other outcomes													
Hyperbilirubinemia	Karaponi Am Okesene-Gafa, 2020	2	RR	0.19	0.06 to 0.61	0	0.522	0.006	20/120	NA	Yes/yes	No	Weak (IV)
Lactation													
Breast milk microbiota													
Detection rate of beneficial bacteria in breast milk	Bekalu Kassie Alemu, 2023	6	RR	2.70	1.22 to 5.96	76.1	0.001	0.014	241/655	0.283 to 25.726	Yes/yes	No	Weak (IV)
Pregnancy and lactation													
Gastrointestinal outcomes													
Infant stool beneficial bacteria abundance	Bekalu Kassie Alemu, 2023	6	SMD	0.89	0.38 to 1.40	91.2	0	0.001	894	−0.944 to 2.726	No/NP	Yes	Weak (IV)
Infant colic	Bekalu Kassie Alemu, 2023	3	RR	0.30	0.16 to 0.57	0	0.481	0.000	45/388	0.16 to 0.57	Yes/no	No	Weak (IV)
Immunologic response													
Eczema	G Zuccotti, 2015	10	RR	0.75	0.57 to 0.97	66.7	0.001	0.029	495/1567	0.326 to 1.710	Yes/no	No	Weak (IV)
Breast milk microbiota and feeding practices													
Detection rate of beneficial bacteria in breastmilk	Bekalu Kassie Alemu, 2023	12	RR	1.80	1.25 to 2.58	69.2	0	0.001	494/1308	0.706 to 4.573	Yes/no	No	Weak (IV)
The mean abundance of beneficial bacteria in breast milk	Bekalu Kassie Alemu, 2023	10	SMD	1.22	0.62 to 1.83	92.8	0	0.000	867	−0.982 to 3.435	Yes/no	Yes	Weak (IV)
The mean abundance of pathogenic bacteria in breast milk	Bekalu Kassie Alemu, 2023	6	SMD	−0.90	−1.49 to −0.31	91.3	0	0.003	745	−2.978 to 1.140	Yes/no	No	Weak (IV)
Variable intervention timing (pregnancy and/or lactation)													
Immunologic response													
Atopic eczema	Shuya Sun, 2022	3	RR	0.68	0.50 to 0.93	0	0.42	0.015	134/626	0.50 to 0.93	No/NP	Yes	Weak (IV)
Atopic dermatitis	Feina Wang, 2023	7	RR	0.58	0.25 to 0.90	74.4	0.001	0.001	NA	0.164 to 2.057	Yes/no	No	Weak (IV)
	Jeffrey Voigt, 2022	6	RR	0.52	0.38 to 0.72	48.6	0.083	0.000	266/1180	0.214 to 1.267	Yes/no	No	Weak (IV)
Eczema	Shuya Sun, 2022	5	RR	0.61	0.44 to 0.85	74.4	0.004	0.003	362/1027	0.190 to 1.952	Yes/no	No	Weak (IV)
Fetal growth and development													
SGA	Sarah J Davidson, 2021	3	RR	0.51	0.30 to 0.87	0	0.605	0.012	59/814	0.30 to 0.87	Yes/NP	No	Weak (IV)

CI, confidence interval; PI, prediction interval; SSE, small study effect; ESB, excess significance bias; LS, largest study with significant effect; RR, relative risk; SMD, standardized mean difference; NP, not provided; SGA, small for gestational age.

Among the RCTs, 7 evaluated fermented milk products, including yogurt, while the rest assessed probiotic supplements. Four trials administered probiotics vaginally, with others using various delivery methods, such as capsules or powder. The entire intervention duration ranged from 1 to 55 weeks (median 16 weeks). The tested organisms included 6 *Bifidobacterium* species (*B. actiregularis*, *B. breve*, *B. infantis*, *B. longum*, 2 strains of *B. bifidum*, and 5 strains of *B. animalis*), 12 *Lactobacillus* species (*L. delbrueckii*, *L. plantarum*, *L. fermentum*, *L. reuteri*, *L. casei*, *L. crispatus*, *L. jensenii*, *L. gasseri*, *L. salivarius*, *L. paracasei*, 2 strains of *L. acidophilus*, and 4 strains of *L. rhamnosus*), 1 *Lactococcus* species (*Lc. lactis*), 1 *Saccharomyces* species (*S. boulardii*), and 1 *Streptococcus* species (*S. thermophilus*), taken as single strains or in combination. Daily probiotic dosages ranged from 5 × 10^6^ to 9 × 10^11^ colony-forming units (CFU) per day. Among the included trials, 3 reported only minimum doses without maximum values (range of reported minima: 1 × 10^8^ to 9 × 10^11^ CFU/day); these formulations typically report guaranteed minimum viable counts rather than exact administered amounts (46–48), 5 did not report any dosage information, and 17 used multi-strain formulations without reporting strain-specific dosages. When summarized by genus, *Lactobacillus*-containing formulations exhibited the widest dosage range (1 × 10^8^ to 1.05 × 10^11^ CFU/day; 37 RCTs), followed by *Bifidobacterium*-based preparations (5 × 10^6^ to 1 × 10^10^ CFU/day; 16 RCTs) and mixed *Lactobacillus-Bifidobacterium* formulations (2 × 10^9^ to 9 × 10^11^ CFU/day; 25 RCTs), whereas *Lactococcus*, *Streptococcus*, and *Saccharomyces* were evaluated in a limited number of trials (7 RCTs) with relatively narrower ranges. Study region data were unavailable for 2 trials; of the remainder, 33 were conducted in Europe, 18 in Asia, 9 in Oceania, and 1 each in South America and Africa. Study regions, probiotic strains, and detailed species- and strain-specific dosages are provided in Supplementary file 5.

The 13 SRs with relevant MAs evaluated 62 specific offspring outcome-level associations. These encompassed fetal growth and development (*n* = 18); outcomes related to preterm premature rupture of membranes (PPROM; *n* = 2); timing of delivery (*n* = 9); mode of delivery (*n* = 5); glucose regulation (*n* = 2); pulmonary function (*n* = 1); perinatal mortality (*n* = 2); postnatal mortality (*n* = 1); breast milk microbiota (*n* = 5); breastfeeding practices (*n* = 1); immunologic (*n* = 7); gastrointestinal outcomes (*n* = 2); and other outcomes (*n* = 7) (Table 3).

Notably, 8 associations included pregnant women with gestational diabetes mellitus (GDM), 2 involved healthy pregnant women, 9 assessed women who were overweight or obese before pregnancy (BMI ≥ 25 kg/m^2^), 5 focused on women with PPROM, 3 assessed women at high risk of allergies, and 1 assessed women with dysglycemia. However, the remaining 34 associations showed no consistency in maternal health status across studies (Supplementary file 6).

#### 3.2.2 Methodological quality of quantitative evidence

Based on AMSTAR 2, of the 13 included reviews, 3 were rated as high methodological quality, 9 as low, and 1 as critically low. Correspondingly, across the 62 offspring health-related outcomes, 52% (10 in pregnancy; 6 in pregnancy and lactation; 1 in lactation; 15 in variable intervention timing) were graded as high, 45% (23 in pregnancy; 3 in pregnancy and lactation; 2 in variable intervention timing) as low, and 3% (2 in variable intervention timing) as critically low. The most frequent sources of bias were the absence of a rationale for the types of primary studies included (e.g., RCTs or observational studies), not reporting funding sources of the primary studies, and a lack of a list of excluded studies with reasons for exclusion (Supplementary file 6).

#### 3.2.3 Certainty of quantitative evidence

According to the GRADE assessment, 5% (*n* = 3) of associations were rated as high certainty, 27% (*n* = 17) as moderate, 37% (*n* = 23) as low, and 31% (*n* = 19) as very low, primarily downgraded due to imprecision. Within these ratings, probiotic supplementation during pregnancy accounted for 8 moderate, 10 low, and 15 very low associations; only 1 association during lactation, which was rated as low certainty; during pregnancy and lactation for 1 high, 4 moderate, 3 low, and 1 very low association; and during variable intervention timings for 2 high, 5 moderate, 9 low, and 3 very low associations (Supplementary file 6).

#### 3.2.4 Credibility grading

According to the grading criteria (Table 2), only 17 outcomes across 62 associations were rated as weak credibility (Table 3), namely 5 during pregnancy, 1 during lactation, 6 during pregnancy and lactation, and 5 during pregnancy and/or lactation. These weak associations were primarily limited by insufficient sample sizes, small-study effects, and the 95% PIs including the null. The remaining offspring outcomes are shown in Supplementary file 6.

#### 3.2.5 Probiotic supplementation during pregnancy

**Figure 2:**
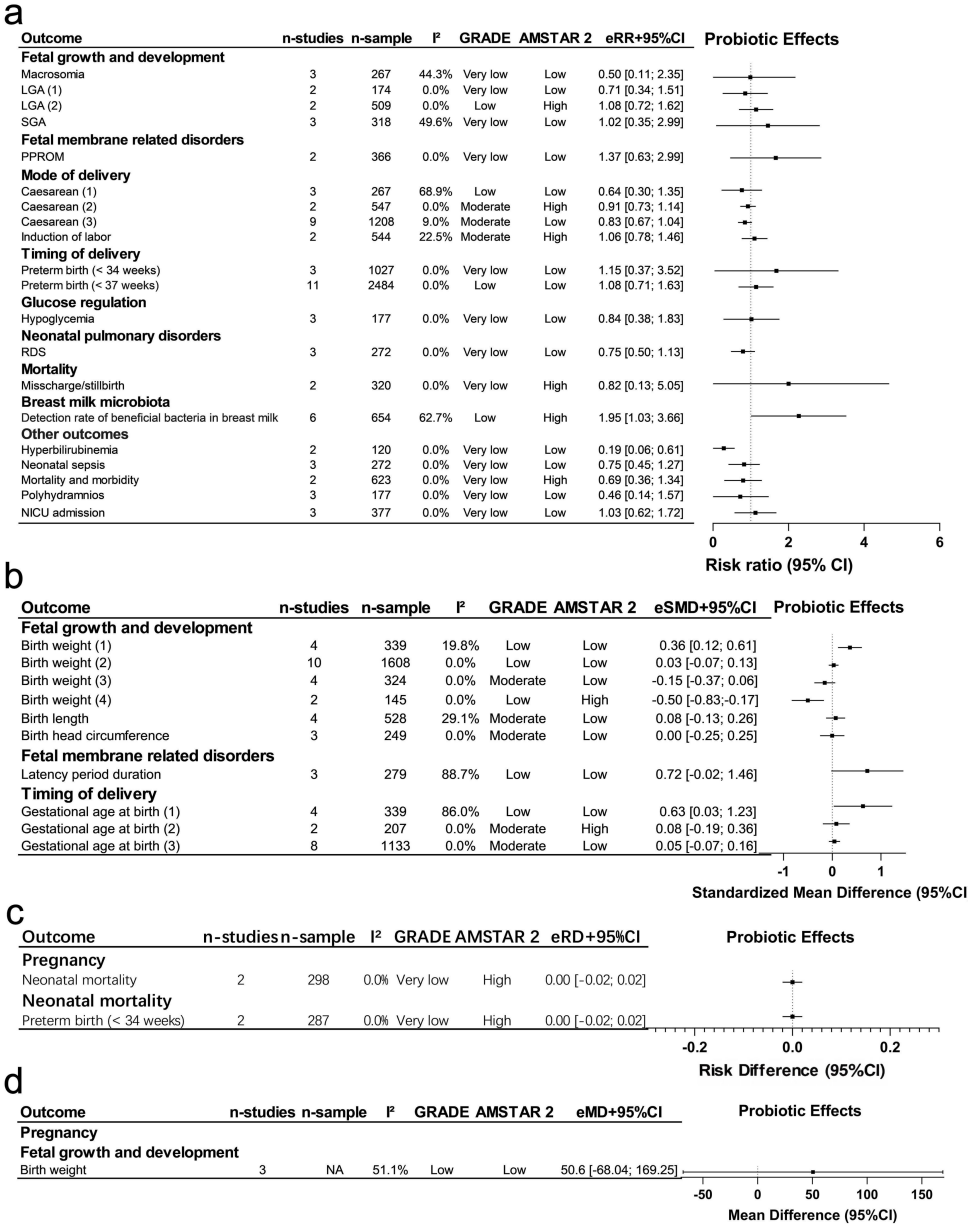
Maternal probiotic supplementation during pregnancy: offspring outcomes, effect size, certainty, and methodological quality. eRR, estimated relative risk; eSMD, estimated standardized mean difference; eMD, estimated mean difference; eRD, estimated risk difference; CI, confidence interval; LGA, large for gestational age; SGA, small for gestational age; PPROM, preterm premature rupture of membrane; RDS, respiratory distress syndrome; NICU, neonatal intensive care unit. **(a)** Categorical outcomes as risk ratios (RR); **(b)** continuous outcomes as standardized mean differences (SMD); **(c)** categorical outcomes as risk differences (RD); **(d)** continuous outcomes as mean differences (MD).

Maternal probiotic supplementation during pregnancy was evaluated across 33 offspring outcome associations, the majority (29 of 33) showed null or modest effects without nominal statistical significance. These findings, together with the corresponding certainty of evidence (8 moderate, 6 low, 14 very low by GRADE) and methodological quality (10 as high, 23 as low, by AMSTAR 2), are presented in Figures 2a–d.

In particular, 5 unique RCT-MAs reported summary results with *p*-values less than 0.05, using random effects (Figures 2a,b). These MAs assessed outcomes such as an increased detection rate of beneficial bacteria in breast milk (*n* = 1), a reduced risk of hyperbilirubinemia (*n* = 1), a slight increase in gestational age (*n* = 1), and variations in birth weight (*n* = 2), showing both increases and decreases. Notably, none of these MAs involved sample sizes exceeding 1,000 participants, which led to weak credibility rating (Class IV) for all 5 outcomes (Table 3). Furthermore, none of the MAs reported a 95% PI excluding the null, and all reported either small-study effects or excess significance. Heterogeneity was not large (*I*^2^ < 50%) for birth weight (with opposing effects), gestational age at birth, or hyperbilirubinemia.

According to GRADE, the certainty of evidence was low for birth weight (with opposing effects), gestational age, and the detection of beneficial bacteria in breast milk, whereas the certainty of evidence for hyperbilirubinemia reduction was very low, with imprecision being the most common reason for downgrading. Low-certainty evidence suggests that the observed changes in birth weight, gestational age, and detection rate of beneficial bacteria should be interpreted cautiously, while very low-certainty evidence indicates substantial uncertainty regarding the reduction in hyperbilirubinemia. Additionally, 3 MAs (birth weight with a positive effect, gestational age at birth, and hyperbilirubinemia) were rated as low quality by AMSTAR 2, failed to report sources of funding of included research, did not adequately investigate publication bias, or did not provide a list of excluded studies with reasons. In contrast, 2 (birth weight with a negative effect and detection of beneficial bacteria in breast milk) were rated as high quality.

#### 3.2.6 Probiotic supplementation during lactation and during both pregnancy and lactation

**Figure 3:**
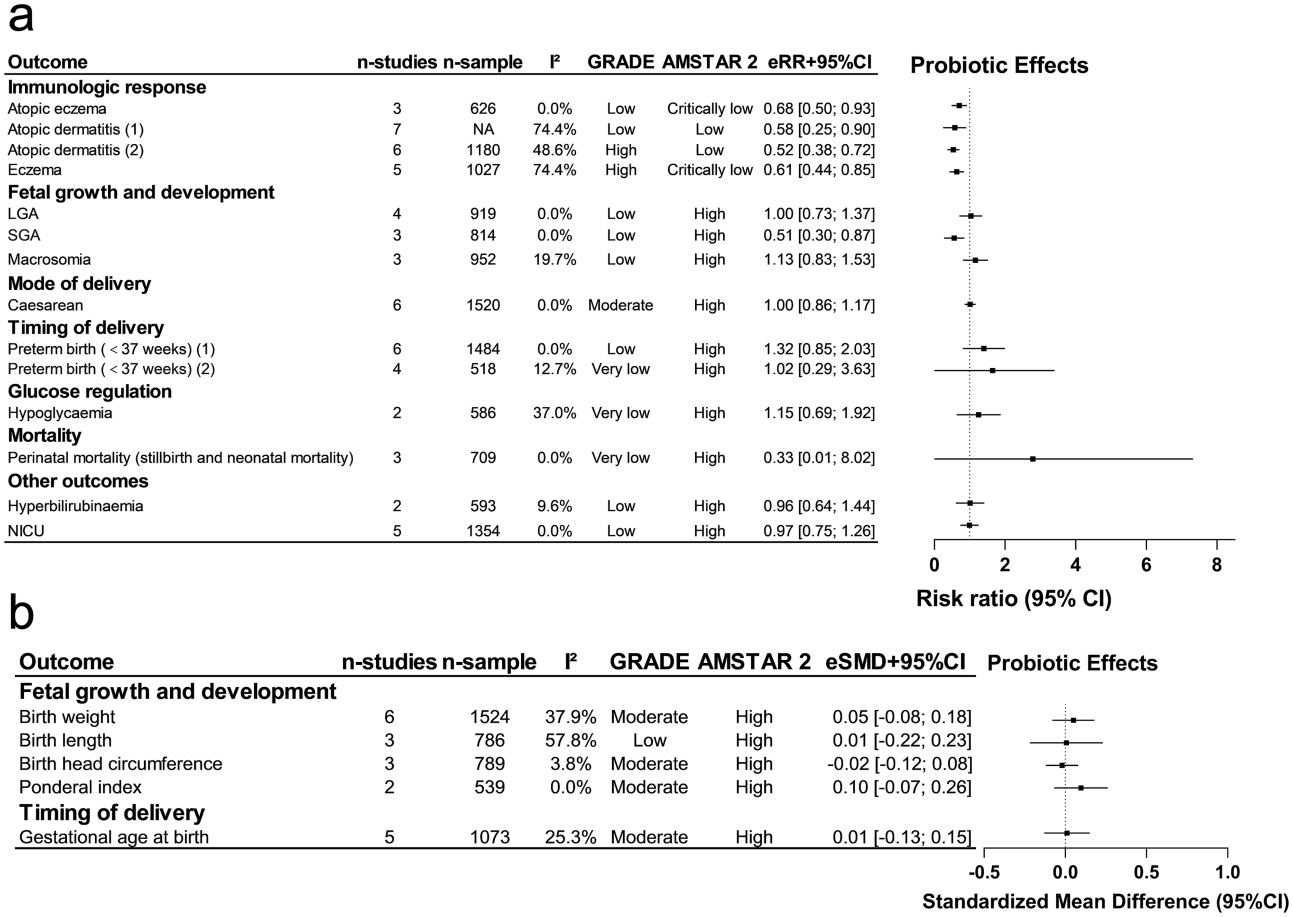
Maternal probiotic supplementation during lactation or pregnancy and lactation: offspring outcomes, effect size, certainty, and methodological quality. eRR, estimated relative risk; eSMD, estimated standardized mean difference; CI, confidence interval. **(a)** Categorical outcomes as risk ratios (RR); **(b)** continuous outcomes as standardized mean differences (SMD).

Due to the limited number of outcomes observed for probiotic supplementation during lactation alone and the combined pregnancy-lactation period, these intervention periods were analyzed together (Figures 3a,b). The detection rate of beneficial bacteria in breast milk was the only outcome exclusively associated with maternal probiotic supplementation during lactation. Consistent with findings from pregnancy-only supplementation (Figure 2a), it demonstrated a positive effect, though the CI was wide because of the small number of events (RR 2.70; 95% CI: 1.22–5.96; 6 studies, 241 events). The certainty of evidence was rated as low per the GRADE system due to inconsistency and imprecision, while methodological quality was rated as high according to AMSTAR 2. Low-certainty evidence may increase in the detection of beneficial bacteria in breast milk. This outcome exhibited very large heterogeneity (*I*^2^ > 75%), and the 95% PI included the null value. Evidence of small-study effects and excess significance was also observed. Consequently, the credibility of this evidence was rated as weak (Table 3).

Nine outcomes were evaluated for maternal probiotic supplementation during both pregnancy and lactation, focusing on breast milk microbiota composition, breastfeeding practices, and gastrointestinal and immunological health. According to GRADE, evidence certainty was rated as high (*n* = 1), moderate (*n* = 4), low (*n* = 3), or very low (*n* = 1). AMSTAR 2 assessments classified 6 outcomes as high quality and 3 as low. Notably, 6 outcomes showed significant associations, including reduced risk of infant colic, lower incidence of eczema, increased abundance of beneficial bacteria in infant stools, and enhanced detection and abundance of beneficial bacteria alongside reduced pathogenic bacteria in breast milk. Of these, 3 outcomes (colic, infant stool beneficial bacteria abundance, and detection rate of beneficial bacteria in breast milk) were rated as low certainty, mainly downgraded for inconsistency and imprecision, 1 (eczema) as high, and 1 (mean abundance of pathogenic bacteria in breast milk) as moderate. High-certainty evidence supports a reduction in eczema risk and moderate-certainty evidence suggests a probable reduction in pathogenic bacteria abundance, whereas the remaining associations should be interpreted cautiously because of low certainty. Regarding quality, only eczema was rated as low, as the review failed to report funding sources, explain the inclusion of RCTs only, or provide a list of excluded studies with reasons, whereas the remaining outcomes were rated high. Eczema and breast milk beneficial bacteria detection involved over 1,000 participants, but only the infant colic outcome had a 95% PI excluding the null. All outcomes were subject to risks of small-study effects and excess significance, with evidence credibility rated as weak for all 6 outcomes (Table 3).

#### 3.2.7 Probiotic supplementation during pregnancy and/or lactation

**Figure 4:**
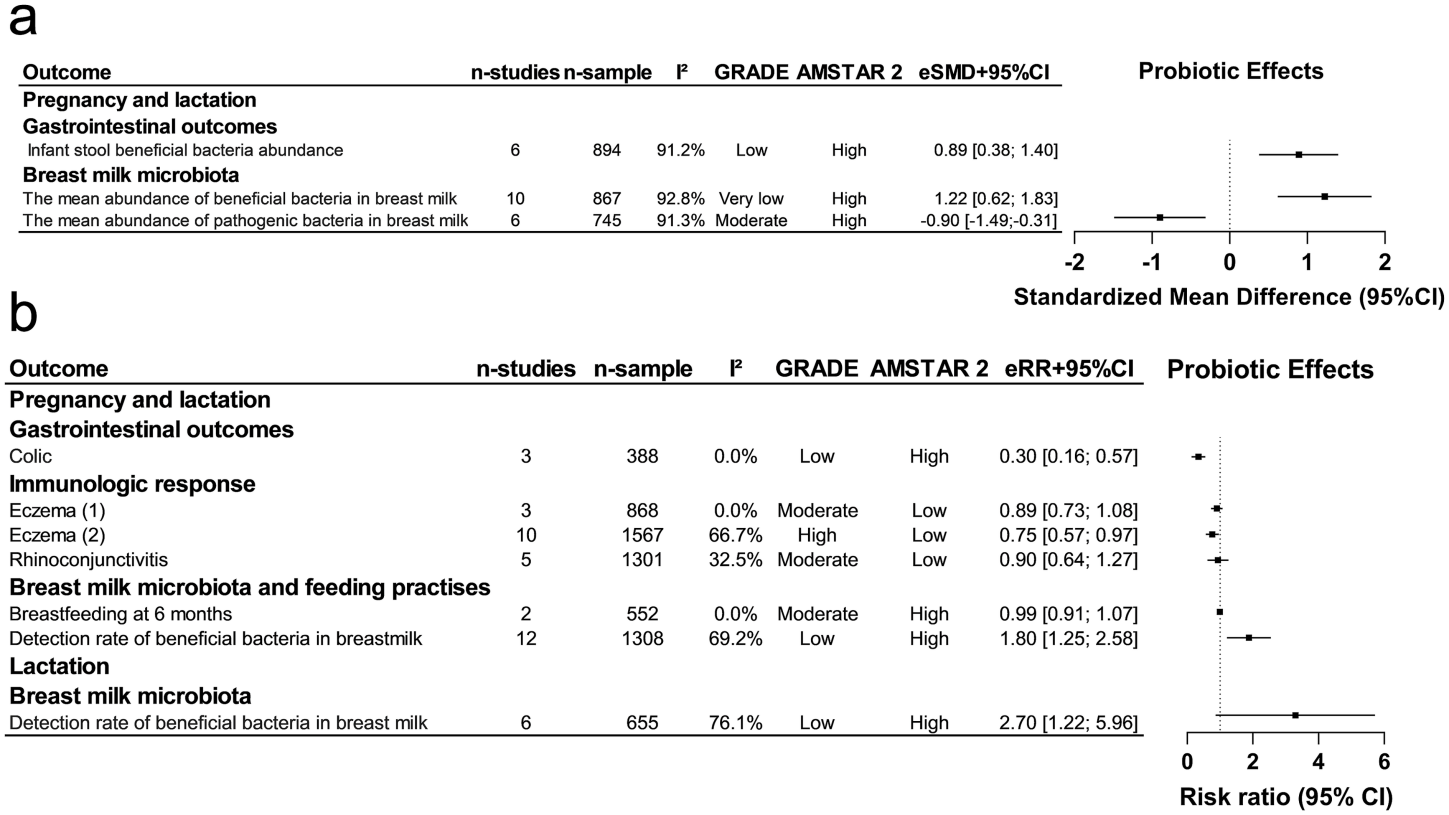
Maternal probiotic supplementation during pregnancy and/or lactation: offspring outcomes, effect size, certainty, and methodological quality. eRR, estimated relative risk; CI, confidence interval; LGA, large for gestational age; SGA, small for gestational age; NICU, neonatal intensive care unit. **(a)** Continuous outcomes as standardized mean differences (SMD); **(b)** categorical outcomes as risk ratios (RR).

Nineteen meta-analytic associations derived from primary studies with interventions during pregnancy, pregnancy and lactation, or lactation, showed varied GRADE evidence levels: high (*n* = 2), moderate (*n* = 5), low (*n* = 9), and very low (*n* = 3). AMSTAR 2 evaluations classified 15 studies as high methodological quality, 2 as low, and 2 as critically low (Figures 4a,b). Most outcomes (14 of 19) showed null or modest effects linked to maternal probiotic supplementation during these variable intervention periods.

Specifically, 5 outcomes demonstrated significant associations (*p* < 0.05 by random effects; Figure 4a), including 3 for a reduced incidence of atopic dermatitis, 1 for eczema, and 1 for small-for-gestational-age (SGA) infants. Of these, 1 atopic dermatitis outcome and the eczema outcome involved over 1,000 participants. Three outcomes (2 atopic dermatitis and 1 SGA) showed no large heterogeneity (*I*^2^ < 50%), with 2 of these (1 atopic dermatitis and SGA) also having 95% PI excluding the null but exhibiting biases: the atopic dermatitis association showed excess significance, while the SGA association showed both small-study effects and excess significance, largely driven by the largest study (with the smallest standard error), which did not show a significant result. Consequently, the credibility of the significant findings was weak (class IV) (Table 3).

According to the GRADE assessment, eczema outcome was rated as high, while SGA was rated as low. For atopic dermatitis, 2 outcomes were rated as low, and 1 was rated as high. Downgrading to low certainty was primarily due to imprecision. High-certainty evidence supports a reduction in eczema and one atopic dermatitis outcome, while the associations for SGA and the other atopic dermatitis outcomes should be interpreted cautiously because of low certainty.

In terms of AMSTAR, SGA was rated as high, while immunological outcomes were rated as low or critically low due to the lack of interpretation for including only RCTs, unclear prospective design, and failure to provide excluded studies with reasons or funding sources of included studies (Figure 4a).

#### 3.2.8 Safety and adverse events

Of the 13 included SRs, 7 inconsistently reported potential adverse events or safety-related pregnancy and offspring outcomes, such as gestational age at birth, preterm birth, PPROM, neonatal hypoglycemia, NICU admission, neonatal sepsis, and perinatal mortality (Supplementary file 6). The remaining SRs focused primarily on preventive efficacy and did not systematically report safety monitoring.

Across the SRs that reported safety outcomes, there was no consistent evidence of an increased risk of serious pregnancy or infant adverse events. Quantitative syntheses showed no statistically significant associations between maternal probiotic supplementation and adverse outcomes, including NICU admission, neonatal sepsis, preterm birth, or PPROM (all *p* > 0.05). However, substantial methodological heterogeneity in safety outcome definitions, incomplete reporting in many trials, and variable follow-up duration limit the ability to draw definitive conclusions regarding rare or long-term harms.

#### 3.2.9 Subgroup analysis

We conducted subgroup analyses for statistically significant associations or those with large heterogeneity (*I*^2^ > 50%), stratified by baseline maternal health status, probiotic genus, dosage, and supplementation duration. These analyses revealed several context-specific significant associations. Health-status stratification showed that beneficial effects were most frequently observed in healthy women and those at allergy risk, with additional associations identified among women with PPROM, above-normal BMI, GDM, or other illnesses. Genus stratification indicated that combined *Lactobacillus* and *Bifidobacterium* and *Lactobacillus* alone accounted for nearly all significant findings, whereas *Bifidobacterium* alone showed none. Dosage patterns suggested that moderate and high doses yielded more significant associations than low doses, with certain effects (e.g., caesarean risk) becoming significant only within certain dosage strata and the combined *Lactobacillus-Bifidobacterium* subgroup. Duration-based stratification showed comparable numbers of significant associations across short, medium, and long intervention periods, with no evident duration-dependent pattern. Heterogeneity remained large (*I*^2^ > 50%) in most initially heterogeneous associations, even after stratification (Supplementary file 7).

#### 3.2.10 Sensitivity analysis

Sensitivity analyses using restricted maximum likelihood (REML) with Hartung-Knapp-Sidik-Jonkman (HKSJ) adjustment were conducted for the 17 significant outcome-level associations (*p* < 0.05) from the primary DL random-effects analyses. This adjustment provides more accurate CI coverage when study numbers are small by accounting for uncertainty in between-study variance (49). Of the 17 associations, 6 (35%) remained significant, including increased infant stool beneficial bacteria abundance, reduced infant colic, increased beneficial bacteria in breast milk during pregnancy and lactation, and reductions in atopic dermatitis and eczema during pregnancy and/or lactation (Supplementary file 8). The remaining 11 associations (65%) lost significance with CIs widening to include the null value, predominantly those with fewer studies and smaller effect sizes.

### 3.3 Narrative synthesis and key findings

Of the eligible offspring outcomes, 30 individual outcome-level associations were based on a single primary study, derived from 9 SRs. One outcome MA originated from a single SR that lacked complete data, specifically the number of events and group sizes for both the experimental and control groups, which prevented extraction. Table 4 presents the key findings of these SRs, which met the UR’s eligibility criteria but were not included in the quantitative synthesis.

**Table 4:** Narrative synthesis of key findings from individual meta-analyses with only one primary study.

First author, year	Key findings
Outcome that included only one primary study	
Pregnancy	
Hania Szajewska, 2018	High-quality evidence suggested that maternal supplementation with LGG only has no statistically significant effect on wheezing or asthma in children followed up to 2 years of age.
Alexander Jarde, 2018	Low-quality evidence suggested that maternal supplementation with *Lactobacillus salivarius* UCC118 for 4 weeks had no significant effect on neonatal ponderal index.
Carlos A Cuello-Garcia, 2015	Moderate-quality evidence suggested that maternal supplementation with LGG did not significantly affect atopic eczema in children aged 6–36 months or overall eczema in those aged 12–24 months.
Guo-Qiang Zhang, 2016	Critically low-quality evidence suggested that maternal supplementation with LGG did not statistically significantly reduce the risk of food sensitization or atopic sensitization in offspring.
Jacquelyn Grev, 2018	High-quality, small-sample evidence suggested that maternal supplementation with mixed probiotics had no significant effect on preterm birth.
Karaponi Am Okesene-Gafa, 2020	Low-quality evidence showed that GDM patients supplemented with *L. salivarius* UCC118 had no significant effect on the induction of labor and cord blood C-peptide levels in neonates.
Lin Li, 2019	Low-quality evidence suggested that maternal LGG supplementation in late pregnancy did not significantly reduce the risk of atopic dermatitis in children at 1 year.
Lactation	
Jacquelyn Grev, 2018	High-quality, small-sample evidence suggested that postnatal maternal supplementation with *L. acidophilus* and *B. lactis* until hospital discharge did not significantly reduce mortality or major complications (ROP, NEC, Sepsis, BPD, PDA, IVH, PVL) in VLBW infants and offered minimal benefit for achieving 50% enteral feeding.
Carlos A Cuello-Garcia, 2015	Moderate-quality evidence suggested that maternal supplementation did not significantly reduce the risk of eczema or allergy in children aged 1–2 years.
Pregnancy and lactation	
Sarah J Davidson, 2021	High-quality, small-sample evidence suggested that maternal supplementation with LGG and *B. lactis* during pregnancy and lactation had no significantly effect on head circumference, height, weight gain, metabolic risk reduction (32–33 split proinsulin levels), and mean blood pressure in infants. And maternal *L. rhamnosus* HN001 supplementation from early pregnancy to delivery showed no significant effect on caesarean section rates.
Jacquelyn Grev, 2018	High-quality, small-sample evidence suggested that maternal supplementation with LGG and *B. lactis* showed no significant reduction in late-onset sepsis during pregnancy and lactation.
Meghan B Azad, 2013	Critically low-quality evidence suggested that maternal supplementation with LGG, *L. acidophilus*, and *B. lactis* from late pregnancy to 3 months postpartum did not significantly reduce asthma risk in children at age 2.
MAs reporting incomplete data of outcome	
Pregnancy and lactation	
Guo-Qiang Zhang, 2016	Maternal probiotic supplementation showed no significant effect on atopic sensitization in infants.

*L.*, *Lactobacillus*; *B.*, *bifidobacterium*; LGG, *Lactobacillus rhamnosus* GG; GDM, gestational diabetes mellitus; ROP, Retinopathy of prematurity; NEC, Necrotizing Enterocolitis; BPD, bronchopulmonary dysplasia; PDA, patent ductus arteriosus; IVH, intraventricular hemorrhage; PVL, periventricular leukomalacia; VLBW, very low birth weight.

## 4 Discussion

In this overview, we synthesized evidence from SRs and MAs evaluating maternal probiotic supplementation across 10 offspring health outcome domains: fetal growth and development, fetal membrane disorders, modes of delivery, timing of delivery, glucose regulation, neonatal pulmonary disorders, neonatal mortality, breastmilk microbiota and feeding practices, immunological response, and gastrointestinal outcomes. Most evidence pertained to supplementation during pregnancy, with limited data available for lactation or combined prenatal and postnatal periods, revealing a significant evidence gap. This disparity underscores the need for targeted research to clarify whether the timing of maternal probiotic exposure differentially impacts offspring outcomes. This gap may result from various challenges in studying lactation and combined prenatal and postnatal interventions, particularly the greater difficulty of monitoring infant health outcomes postnatally (50) compared with prenatally, exacerbated by fewer routine clinical visits for postpartum women (51), which restricts timely data collection. Additionally, the heterogeneity of breastfeeding patterns complicates intervention standardization (52), potentially undermining adherence and reliability of outcomes. While advancements in high-throughput sequencing have enhanced breast milk microbiota research, methods for accurately characterizing its composition and function remain under development. Intergenerational studies on the effects of maternal probiotic supplementation remain limited, with the included SRs solely based on RCTs. These feasibility and methodological constraints highlight the urgent need for further studies to address these evidence gaps and refine approaches to investigating maternal probiotic interventions across diverse supplementation windows.

Overall, maternal probiotic supplementation showed statistically significant associations with 17 offspring outcomes. However, only four outcomes (two associations for reduction in eczema, one for atopic dermatitis, and one for pathogenic bacteria in breast milk) demonstrated moderate or high certainty according to GRADE, with none reaching suggestive credibility; all were graded as weak. This indicates that these statistically significant associations demonstrated methodological fragility, with small-study effects, wide PIs including the null, and excess significance bias. Sensitivity analyses using REML with HKSJ adjustment confirmed this fragility, with most associations losing significance when more conservative statistical methods were applied to account for uncertainty in between-study variance. The few remaining significant associations were primarily related to eczema and breast milk bacteria outcomes, which may represent the most reliable biological signals for further mechanistic investigation. Unlike GRADE, which assesses internal validity via risk of bias, precision, and consistency, credibility grading incorporates additional robustness metrics specific to URs, including PIs, small-study effects, and excess significance testing, making it more conservative and better suited to evaluate reproducible effects. In our analyses, the 95% PIs often included the null due to large heterogeneity and limited sample sizes (53). Heterogeneity was inherited from the included MAs, likely stemming from baseline population differences, methodological inconsistencies, and study design variations (54), contributing to wider CIs (55). We attempted to further refine strain-level stratification to mitigate heterogeneity, but this was not feasible due to mixed probiotic interventions across studies. Additionally, small-study effects were also evident when the largest, most precise study reported null results while several smaller ones showed significant effects, potentially inflating pooled estimates. Although Egger’s test detected no publication bias, its low power with few studies means that bias cannot be excluded (56). These factors collectively drove excess significance bias (26), though some MAs did not exhibit this bias due to differences in design or baseline characteristics between smaller studies and the largest study. Taken together, while maternal probiotic supplementation shows potential links to certain offspring outcomes, the reliability of these findings remains limited, warranting cautious interpretation. Larger, well-designed trials and standardized SRs are required to validate and strengthen the evidence base.

Given the persistent but weak signals in sensitivity analyses, we focus on mechanistic pathways to interpret associations of moderate-to-high certainty, such as the reduced risk of eczema and atopic dermatitis and improved breast milk microbiota composition, while exploring their underlying causes.

Multiple clinical trials have indicated that maternal probiotic supplementation may offer protective effects against eczema development in offspring through various mechanistic pathways (57, 58). Probiotics promote colonization of beneficial bacteria in the maternal gut, vagina, and breast milk microbiota (35, 42, 59), which shapes the newborn’s microbiome (59–61). This aligns with our finding of reduced pathogenic bacteria in breast milk associated with supplementation. Furthermore, prenatal probiotics may also enhance placental immune activity and increase IFN-*γ* in cord blood (62), supporting fetal immune balance. Through the entero-mammary pathway, supplementation may upregulate regulatory T-cell responses and increase TGF-β2 in breast milk, contributing to immune tolerance in newborns (63). However, as shown in previous trials, prenatal supplementation alone may be insufficient; combined prenatal and postnatal supplementation may be required to modulate key immune markers such as IgA and sCD14 (64). Our UR similarly found no significant reduction in eczema in analyses restricted to prenatal-only interventions.

The subgroup analyses provide further insights into these mechanistic pathways. Significant effects were most consistently observed in women at risk of allergies, including reductions in eczema and atopic dermatitis, plausibly due to heightened baseline immune dysregulation that may increase responsiveness to probiotic-mediated immunomodulation (65). Distinct patterns among women with PPROM, above-normal BMI, GDM, or other illnesses further suggest that probiotic efficacy may depend on maternal physiology rather than acting uniformly across populations. Probiotic composition appeared to influence outcomes; combinations of *Lactobacillus* and *Bifidobacterium* yielded the most significant associations, whereas *Bifidobacterium* alone showed none, supporting potential synergistic actions along the gut-immune-mammary axis. Furthermore, moderate to high dosages (>10^9^ CFU/day) generally produced greater benefits than low dosages, consistent with biological expectations. Additionally, some outcomes also appeared responsive even to relatively short-term supplementation, indicating that certain probiotic effects may emerge within relatively brief exposure windows. Notably, multi-genus formulations at moderate dosages significantly reduced caesarean risk despite the overall non-significant effect, indicating a synergistic benefit of adequately dosed multi-genus regimens. However, a few associations (improved beneficial bacteria in breast milk) became non-significant after stratification, likely reflecting the fragility of some effects due to reduced statistical power after stratification. Despite subgrouping, heterogeneity remained large (*I*^2^ > 50%) in most associations that initially showed large variability, underscoring the multifactorial and complex nature of probiotic actions. Variations in strains, study designs, maternal and infant characteristics, diagnostic criteria or outcome definitions, and substantial heterogeneity in microbiome composition likely contribute to this complexity, reducing the stability and precision of pooled estimates, limiting generalizability, and complicating the interpretation of associations.

Critical evidence gaps persist, limiting confident clinical application. Although RCTs report no consistent adverse events, current data cannot exclude rare harms or establish safety in vulnerable subgroups, warranting caution. Essential intervention parameters, including optimal strains, dose, and timing, remain undefined, and the limited effects of lactation-only regimens suggest, but do not confirm, the primacy of prenatal microbial priming. Additionally, route of administration represents an under-explored dimension: only 1 included SR (42) evaluated vaginal probiotic administration (4 RCTs, all in women with PPROM), precluding direct comparison with oral supplementation. Given the distinct mechanisms whereby vaginal probiotics may influence neonatal microbiome acquisition via birth canal colonization (66), dedicated comparative studies are needed to elucidate route-specific effects. Even the most promising associations (e.g., reduced eczema risk) rest on weak credibility. Thus, clinicians should adopt shared decision-making, communicating modest potential benefits for select maternal groups alongside substantial uncertainty. For women choosing probiotics, multi-genus formulations at adequate doses may be reasonable, but individual variability and evidence limitations must be emphasized.

Overall, while maternal probiotic supplementation during pregnancy and lactation shows promise, notably in reducing eczema risk for high-risk populations and decreasing pathogenic bacteria in breast milk, the evidence remains constrained by methodological inconsistencies, moderate certainty, and variable credibility. The lack of robustness observed in sensitivity analyses suggests that most observed associations may not yet be fully reproducible but instead reflect artifacts arising from methodological limitations in the primary evidence base. More rigorously designed trials and well-defined SRs are needed, incorporating stricter baseline health criteria, dietary confounder controls, refined probiotic strains, multi-center/multi-ethnic designs, varying pregnancy stages, and extended follow-ups, to clarify strain-, dose-, and population-specific effects, as well as long-term maternal and offspring safety outcomes, thereby enabling larger-scale studies.

## 5 Strengths and limitations

Our UR with re-analysis synthesizes evidence on the effects of maternal probiotic supplementation during pregnancy, lactation, or both on offspring health-related outcomes. Data were extracted from each included study, and summary effect sizes, along with other indicators, were recalculated. The evidence was systematically evaluated for methodological quality, certainty, and credibility. Outcomes were primarily analyzed quantitatively, with qualitative synthesis for less-reported outcomes. These findings provide insights for clinical and public health guidance while highlighting research gaps.

Our review also had several limitations. First, as an UR, it focuses on existing MAs and thus may exclude outcomes without such analyses, including rare offspring health effects. Although no restriction on primary study design was imposed at the eligibility stage, all SRs that met our inclusion criteria were based exclusively on RCTs. This limited the overall sample size. Additionally, for small-sample studies, the DL random-effects model may yield narrower 95% CIs, potentially inflating statistical significance and reducing reliability (67). Our sensitivity analyses using REML with HKSJ adjustment showed that 65% of initially significant associations lost significance, underscoring the need for caution when interpreting findings based on few studies. Second, focusing on maternal-offspring transmission excluded studies of direct infant probiotic administration. Furthermore, many MAs focused on secondary outcomes, introducing heterogeneity and weakening certainty and credibility (54). Lastly, variations in complementary feeding and non-exclusive breastfeeding may have contributed to inconsistent lactation-related outcomes.

## 6 Conclusion

In conclusion, although maternal probiotic supplementation during pregnancy or lactation showed statistical associations with eczema risk and breast milk microbiota, the credibility of current evidence is weak because of insufficient sample sizes, small-study effects, excess significance bias, heterogeneity, and methodological limitations. These findings do not support clinical recommendations. Further well-designed, large-scale studies are likely to significantly advance our understanding in this field.

## Data Availability

The original contributions presented in the study are included in the article/Supplementary material, further inquiries can be directed to the corresponding author.

## Footnotes

1
https://www.crd.york.ac.uk/PROSPERO/view/CRD420251120972

2
https://gradepro.org/
