# Supplementary material for: Maternal probiotic supplementation and offspring health: an umbrella review with re-analysis of systematic reviews and meta-analyses
Source: Front Nutr. 2026 Mar 26;13:1764109. doi: 10.3389/fnut.2026.1764109 (PMC13062327; doi:10.3389/fnut.2026.1764109)
Supplement: Supplementary file 2 [file Table_2.DOCX]

**Supplementary materials**

**Supplement 1.** Search strategy and keywords for each database

**Supplement 4.** Reasons for the exclusion of reviews that were fully accounted for

GRADE ratings of included associations between maternal probiotic supplementation and offspring outcomes

Supplement 1. Search strategy and keywords for each database

**Search terms for each database**

| **PubMed** |
| --- |
| **MeSH terms**  Systematic Review [Publication Type]  Meta-Analysis [Publication Type]  Mothers  Pregnant People  Pregnancy  Lactation  Breast Feeding  Probiotics  Dietary Supplements  Infant  Infant, Newborn  **Entry terms**  **Systematic Review [Publication Type]**  Review, Systematic  Systematic review  **Meta-Analysis [Publication Type]**  Meta analysis  **Mothers**  Mother  **Pregnant People**  People, Pregnant  Pregnant Peoples  Pregnant Person  Pregnant Woman  Woman, Pregnant  Women, Pregnant  Pregnant Women  **Pregnancy**  Pregnancies  Gestation  **Lactation**  Milk Secretion*  Lactation*, Prolonged  Prolonged Lactation*  **Breast Feeding**  Breast Feedings  Breast Fed  Breastfed  Chestfeeding*  Breastfeeding  Breast Feeding, Exclusive  Exclusive Breast Feeding  Breastfeeding, Exclusive  Exclusive Breastfeeding  Wet Nursing  Milk Sharing  Sharing, Milk  **Probiotics**  Probiotic  **Dietary Supplements**  Dietary Supplement  Supplements, Dietary  Dietary Supplementations  Supplementations, Dietary  Food Supplement*  Supplement*, Food  Nutraceutical*  Nutriceutical*  Neutraceutical*  Herbal Supplement*  Supplement*, Herbal  **Infant**  Infants  **Infant, Newborn**  Infants, Newborn  Newborn Infant*  Neonate  Neonates  Newborns  Newborn  **Search string**  #1  "Systematic Review"[Publication Type] OR "review systematic"[Title/Abstract] OR "Systematic Review"[Title/Abstract] OR "Meta-Analysis"[Publication Type] OR "Meta analysis"[Title/Abstract]  #2  "Mothers"[MeSH Terms] OR "Mother"[Title/Abstract] OR "Pregnant People"[MeSH Terms] OR "People, Pregnant"[Title/Abstract] OR "Pregnant Peoples"[Title/Abstract] OR "Pregnant Person"[Title/Abstract] OR "Pregnant Woman"[Title/Abstract] OR "Woman, Pregnant"[Title/Abstract] OR "Women, Pregnant"[Title/Abstract] OR "Pregnant Women"[Title/Abstract] OR "Pregnancy"[MeSH Terms] OR "Pregnancies"[Title/Abstract] OR "Gestation"[Title/Abstract] OR "Lactation"[MeSH Terms] OR "Milk Secretion*"[Title/Abstract] OR "Lactation*, Prolonged"[Title/Abstract] OR "Prolonged Lactation*"[Title/Abstract] OR "Breast Feeding"[MeSH Terms] OR "Breast Feedings"[Title/Abstract] OR "Breast Fed"[Title/Abstract] OR "Breastfed"[Title/Abstract] OR "Chestfeeding*"[Title/Abstract] OR "Breastfeeding"[Title/Abstract] OR "Breast Feeding, Exclusive"[Title/Abstract] OR "Exclusive Breast Feeding"[Title/Abstract] OR "Breastfeeding, Exclusive"[Title/Abstract] OR "Exclusive Breastfeeding"[Title/Abstract] OR "Wet Nursing"[Title/Abstract] OR "Milk Sharing"[Title/Abstract] OR "Sharing, Milk"[Title/Abstract]  OR "Perinatal"[Title/Abstract] OR "Maternal"[Title/Abstract]  #3  "Probiotics"[MeSH Terms] OR "Probiotic"[Title/Abstract] OR "Dietary Supplements"[MeSH Terms] OR "Dietary Supplement"[Title/Abstract] OR "Supplements, Dietary"[Title/Abstract] OR "Food Supplement*"[Title/Abstract] OR "Supplement*, Food"[Title/Abstract] OR "Nutraceutical*"[Title/Abstract] OR "Nutriceutical*"[Title/Abstract] OR "Neutraceutical*"[Title/Abstract] OR "Herbal Supplement*"[Title/Abstract] OR "Supplement*, Herbal"[Title/Abstract] OR "Nutritional Supplement"[Title/Abstract]  #4  "Infant"[MeSH Terms] OR "Infants"[Title/Abstract] OR "Infant, Newborn"[MeSH Terms] OR "Infants, Newborn"[Title/Abstract] OR "Newborn Infant*"[Title/Abstract] OR "Neonate"[Title/Abstract] OR "Neonates"[Title/Abstract] OR "Newborns"[Title/Abstract] OR "Newborn"[Title/Abstract] OR "Offspring"[Title/Abstract] OR "Baby"[Title/Abstract]  #1 AND #2 AND #3 AND #4 |
| **Web of Science** |
| MeSH terms were the same as those used in PubMed.  Entry terms were the same as those in PubMed.  The search string was the same as the one in PubMed. |
| **Embase** |
| **Emtree and entry terms**  **systematic review (#1 Emtree are medical subjective headings, and entry terms were restricted to titles or abstracts and combined with Emtree using ‘OR’)**  'review, systematic' OR 'systematic review'  **Meta analysis #2**  'analysis, meta' OR 'meta-analysis' OR 'metaanalysis' OR 'meta analysis'  **Mother #3**  'motherhood' OR 'mothering' OR 'mothers' OR 'mother' OR ‘maternal’  **Pregnant person #4**  'pregnant people' OR 'pregnant persons' OR 'pregnant person' OR ‘people, pregnant’ OR ‘pregnant peoples’ OR ‘pregnant woman’ OR ‘Woman, pregnant’ OR ‘Women pregnant’ OR ‘Pregnant Women’  **Pregnancy #5**  'child bearing' OR 'childbearing' OR 'gestation' OR 'gravidity' OR 'intrauterine pregnancy' OR 'labor presentation' OR 'labour presentation' OR 'pregnancy maintenance' OR 'pregnancy trimesters' OR 'pregnancy' OR ‘pregnancies’ OR ‘perinatal’  **Lactation #6**  'breast secretion' OR 'lactic secretion' OR 'mammary gland secretion' OR 'milk excretion' OR 'milk release' OR 'milk secretion' OR 'lactation' OR ‘milk secretion*’ OR ‘lactation*, prolonged’ OR ‘prolonged lactation*’  **Breast feeding #7**  'feeding, breast' OR 'breast feeding' OR ‘breast feedings’ OR ‘breast fed’ OR ‘breastfed’ OR ‘chestfeeding*’ OR ‘breastfeeding’ OR ‘breast feeding, exclusive’ OR ‘exclusive breast feeding’ OR ‘breastfeeding, exclusive’ OR ‘exclusive breastfeeding’ OR ‘wet nursing’ OR ‘milk sharing’ OR ‘sharing, milk’  **Probiotic agent #8**  'probiotic' OR 'probiotics' OR 'probiotic agent'  **Dietary supplement #9**  'diet additive' OR 'diet supplement' OR 'dietary supplements' OR 'food supplement' OR 'supplementary diet' OR 'dietary supplement' OR ‘supplements, dietary’ OR ‘dietary supplementations’ OR ‘supplementations, dietary’ OR ‘food supplement*’ OR ‘supplement*, food’ OR ‘nutraceutical*’ OR ‘nutriceutical*’ OR ‘neutraceutical*’ OR ‘herbal supplement*’ OR ‘supplment*, herbal’  **Nutrition supplement #10**  'enteral nutrition product' OR 'nutrition mixture' OR 'nutrition support product' OR 'nutritional mixture' OR 'nutritional supplement' OR 'parenteral nutrition product' OR 'nutrition supplement'  **Infant #11**  'infant' OR ‘infants’ OR ‘offspring’  **Baby #12**  ‘baby’  **Newborn #13**  'child, newborn' OR 'full term infant' OR 'human neonate' OR 'human newborn' OR 'infant, newborn' OR 'neonate' OR 'neonatus' OR 'newborn baby' OR 'newborn child' OR 'newborn infant' OR 'newly born baby' OR 'newly born child' OR 'newly born infant' OR 'newborn' OR ‘infant, newborn’ OR ‘infants, newborn’ OR ‘newborn, infant*’ OR ‘neonates’ OR ‘newborns’  **Search string**  (#1 OR #2) AND (#3 OR #4 OR #5 OR #6 OR #7) AND (#8 OR #9 OR #10) AND (#11 OR #12 OR #13) |
| **CINAHL** |
| MeSH terms were the same as those used in PubMed.  Entry terms were the same as those in PubMed.  Entry terms were restricted to titles or abstracts and combined with MeSH terms using ‘OR’.  The search string was the same as the one in PubMed. |
| **Cochrane Library** |
| **MeSH terms**  Systematic review  Meta analysis  Breast feeding  Lactation  Pregnancy  Probiotics  Infant, newborn  Infant  Dietary supplement  Mothers  Pregnant people  **Entry terms**  **Systematic review (#1 Entry terms are the same as those in PubMed and restricted to abstracts or titles and combined with MeSH terms using ‘OR’)**  Review, Systematic  **Meta analysis #2**  **Breast feeding #3**  Breast Feedings  Breast Fed  Breastfed  Chestfeeding*  Breastfeeding  Breast Feeding, Exclusive  Exclusive Breast Feeding  Breastfeeding, Exclusive  Exclusive Breastfeeding  Wet Nursing  Milk Sharing  Sharing, Milk  **Lactation #4**  Milk Secretion*  Lactation*, Prolonged  Prolonged Lactation*  **Pregnancy #5**  Pregnancies  Gestation  **Probiotics #6**  Probiotic  **Infant, newborn #7**  Infants, Newborn  Newborn Infant*  Neonate  Neonates  Newborns  Newborn  **Infant #8**  Infants  **Dietary supplement #9**  Dietary Supplement*  Supplement*, Dietary  Food Supplement*  Supplement*, Food  Nutriceutical*  Nutraceutical*  Neutraceutical*  Herbal Supplement*  Supplement*, Herbal  **Mothers #10**  Mother  **Pregnant people #11**  Women, Pregnant  Woman, Pregnant  Pregnant Wom*  People, Pregnant  Pregnant Pe*  **Search string**  (#1 OR #2) AND (#3 OR #4 OR #5 OR #10 OR #11) AND (#6 OR #9) AND (#7 OR #8) |

| Supplement 4. Reasons for the exclusion of reviews that were fully accounted for |
| --- |
| **Reasons for the exclusion of reviews that were fully accounted for** |
| **Interventions include prebiotics or synbiotics** |
| **Title** |
| 1. Carbohydrate supplementation of human milk to promote growth in preterm infants. 2. Effects of Probiotics on Glycemic Control and Metabolic Parameters in Gestational Diabetes Mellitus: Systematic Review and Meta-Analysis. 3. Prebiotics for the prevention of allergies: A systematic review and meta-analysis of randomized controlled trials. 4. Probiotics and prebiotics: Clinical effects in allergic disease. 5. Probiotics and synbiotics show clinical efficacy in treating gestational diabetes mellitus: A meta-analysis. 6. Probiotics to prevent infantile colic. 7. The effect of probiotics added to maternal nutrition on infantile colic: A systematic review and meta-analysis. |
| **Comprehensive dietary intervention that confounder with other nutritional ingredient or food** |
| **Title** |
| 1. Diet or exercise, or both, for preventing excessive weight gain in pregnancy. 2. Dietary exposures during pregnancy, lactation or infancy and risk of allergic diseases: A systematic review and meta-analysis. 3. Early-Life Nutrition Interventions and Associated Long-Term Cardiometabolic Outcomes: A Systematic Review and Meta-Analysis of Randomized Controlled Trials. 4. Effects of dietary interventions on neonatal and infant outcomes: a systematic review and meta-analysis. 5. Effects of dietary interventions on pregnancy outcomes: a systematic review and meta-analysis. 6. Interventions for preventing excessive weight gain during pregnancy. 7. Maternal weight status, diet, and supplement use as determinants of breastfeeding and complementary feeding: a systematic review and meta-analysis. 8. Participant attrition and perinatal outcomes in prenatal vitamin D-supplemented gestational diabetes mellitus patients in Asia: A meta-analysis. 9. Systematic review of clinical trials on dietary interventions to prevent excessive weight gain during pregnancy among normal weight, overweight and obese women. |
| **No-English reviews** |
| **Title** |
| 1. Atopic dermatitis. Probiotics in primary prevention. 2. Effect of early probiotic intervention effect in preventing allergic diseases in children: a meta analysis. 3. Effect of probiotic supplementation during pregnancy and infancy in preventing atopic dermatitis in children: A Meta analysis. 4. Efficacy and effectiveness of 20 child health interventions in China: Systematic review of Chinese literature. 5. Living preparation of lactobacillus versus metronidazole for bacterial vaginosis in pregnancy: A systematic review. 6. Meta-analysis of probiotics preventing allergic diseases in infants. 7. Metaanalyse zu präventiven und therapeutischen Effekten probiotischer Supplementierung bei Kindern mit atopischer Dermatitis. 8. Nutrition and physical activity in infants and breastfeeding women 2024 Partly updated recommendations by "Healthy Start Network" (Netzwerk Gesund ins Leben). 9. Preventative effect of probiotics for infantile atopic dermatitis: A systematic review. 10. Research update on the effects of probiotic supplementation during pregnancy on pregnancy complications: a systematic literature review. 11. Systematic review: Dietary treatment of infant colic (and II). 12. Systematic review: Dietary treatment of infant colic (I). 13. What's new in the evidence-based and consented guideline on allergy prevention? |
| **Not peer-reviewed reviews** |
| **Title** |
| 1. An in-depth investigation into the associations between maternal dietary intake during pregnancy and obesity and allergy outcomes in children. 2. Is Probiotic Supplementation During Pregnancy Safe for Healthy Populations? A Systematic Review of Preclinical Studies. |
| **Publications type are not systematic reviews or meta-analyses** |
| **Title** |
| 1. A systematic review and meta-analysis of participant characteristics in the prevention of gestational diabetes: a summary of evidence for precision medicine. 2. An Early-customized Low Glycaemic-index (GI) Diet Prevents LGA Babies in Overweight/Obese Pregnant Women. 3. An overview of systematic reviews of complementary and alternative therapies for infantile colic. 4. An Overview of Systematic Reviews of Randomized-Controlled Trials for Preventing Necrotizing Enterocolitis in Preterm Infants. 5. Are maternally administered probiotic strains transferred to breast milk: A systematic review of human studies. 6. Are probiotics safe for use during pregnancy and lactation? 7. Associations of Maternal Nutritional Status and Supplementation with Fetal, Newborn, and Infant Outcomes in Low-Income and Middle-Income Settings: An Overview of Reviews. 8. Characteristics of randomized controlled trials included in systematic reviews of nutritional interventions reporting maternal morbidity, mortality, preterm delivery, intrauterine growth restriction and small for gestational age and birth weight outcomes. 9. Community-based supplementary feeding for food insecure, vulnerable and malnourished populations - an overview of systematic reviews. 10. Effects of maternal nutritional supplements and dietary interventions on placental complications: An umbrella review, meta-analysis and evidence map. 11. Gastrointestinal Microbiome and Multiple Health Outcomes: Umbrella Review. 12. Human milk oligosaccharide profiles and associations with maternal nutritional factors: A scoping review. 13. Impact of packaged interventions on neonatal health: a review of the evidence. 14. Interventions other than psychosocial, psychological and pharmacological interventions for preventing postpartum depression: A Cochrane systematic review. 15. Maternal probiotic supplementation for prevention of morbidity and mortality in preterm infants. 16. Mechanisims of asthma and allergic disease-1075. Probiotics in infants for prevention of allergic disease and food hypersensitivity. 17. Microbiota-targeted interventions and clinical implications for maternal-offspring health: An umbrella review of systematic reviews and meta-analyses of randomised controlled trials. 18. Optimal mode of delivery for using probiotics or prebiotics to prevent eczema: A systematic review and meta-analysis. 19. Prevention of Recurrent Spontaneous Preterm Delivery Using Probiotics (Clostridium butyricum, Enterococcus faecium, and Bacillus subtilis; PPP Trial): Protocol for a Prospective, Single-Arm, Nonblinded, Multicenter Trial. 20. Probiotics and Allergy. 21. Probiotics in women with gestational diabetes: Impact on pregnancy and metabolism. 22. The Effect of Probiotics Added to the Mother's Diet on the Occurrence of Necrotizing Enterocolitis and Sepsis and Other Parameters in Preterm Babies. 23. The effects of prebiotic, probiotic or synbiotic supplementation on overweight/obesity indicators: an umbrella review of the trials' meta-analyses. 24. The Efficacy of Probiotics to Reduce Antepartum Group B Streptococcus Colonization. 25. The future of Cochrane Neonatal. 26. The impact of prebiotics, probiotics and synbiotics on the prevention and treatment of atopic dermatitis in children: an umbrella meta-analysis. 27. The role of gut microbiota in mediating allergic asthma in infants. 28. Treatment of the Intermediate Vaginal Microbiota With Vaginal Probiotics Containing Lactobacillus Casei Rhamnosus (Lcr Regenerans) to Improve Pregnancy Outcomes. 29. World Allergy Organization-McMaster University Guidelines for Allergic Disease Prevention (GLAD-P): Prebiotics. 30. World Allergy Organization-McMaster University Guidelines for Allergic Disease Prevention (GLAD-P): Probiotics. 31. A systematic review investigating maternal supplementation with a probiotic during pregnancy and neonatal gut colonization with that specific probiotic. 32. Bacterial munch for infants: Potential pediatric therapeutic interventions of probiotics. 33. Bugs and Guts: Practical Applications of Probiotics for Gastrointestinal Disorders in Children. 34. Childhood Obesity: Current Situation and Future Opportunities. 35. Clinical effects of probiotics: scientific evidence from a paediatric perspective. 36. Dysbiosis and Prematurity: Is There a Role for Probiotics? 37. EAACI food allergy and anaphylaxis guidelines. Primary prevention of food allergy. 38. Early life factors, diet and microbiome, and risk of inflammatory bowel disease. 39. Efficacy of dietary supplements targeting gut microbiota in the prevention and treatment of gestational diabetes mellitus. 40. Gut microbiota and allergic disease: new findings. 41. Impact of the environment on gut microbiome and allergy. 42. Is asthma prevention possible with dietary manipulation? 43. Maternal gut microbiota in the health of mothers and offspring: from the perspective of immunology. 44. Maternal microbe-specific modulation of the offspring microbiome and development during pregnancy and lactation. 45. Microbial regulation of offspring diseases mediated by maternal-associated microbial metabolites. 46. New Insights Into Microbiota Modulation-Based Nutritional Interventions for Neurodevelopmental Outcomes in Preterm Infants. 47. No consistent evidence to date that prenatal or postnatal probiotic supplementation prevents childhood asthma and wheeze. 48. Overview of evidence in prevention and aetiology of food allergy: A review of systematic reviews. 49. Preterm birth: A narrative review of the current evidence on nutritional and bioactive solutions for risk reduction. 50. Prevention of atopic dermatitis. 51. Probiotic Lactobacillus rhamnosus species: considerations for female reproduction and offspring health. 52. Probiotic Supplementation during Pregnancy: Evaluating the Current Clinical Evidence against Gestational Diabetes Mellitus. 53. Probiotics and vaginal microecology: Fact or fancy? 54. Probiotics for allergy prevention. 55. Probiotics for the prevention of atopic dermatitis and other allergic diseases: What are the real facts? 56. Probiotics for treatment and primary prevention of allergic diseases and asthma: looking back and moving forward. 57. Probiotics in perinatal medicine. 58. Probiotics in the Prevention of Necrotizing Enterocolitis. 59. Rationale of Probiotic Supplementation during Pregnancy and Neonatal Period. 60. Recent knowledge of the impact of antenatal probiotic supplementations on the risk of obesity in offspring: A review of literature 61. Role of human microbiota in skin dermatitis and eczema: A systematic review 62. S3 guideline Allergy Prevention. 63. S3-Guideline on allergy prevention: 2014 update: Guideline of the German Society for Allergology and Clinical Immunology (DGAKI) and the German Society for Pediatric and Adolescent Medicine (DGKJ). 64. Scope and quality of Cochrane reviews of nutrition interventions: a cross-sectional study. 65. Strategies to Prevent Early and Late-Onset Group B Streptococcal Infection via Interventions in Pregnancy. 66. Systematic Reviews to Inform Practice, March/April 2022. 67. The benefits of probiotic interventions in maternal-fetal health: An appraise review. 68. The effect of prenatal and postnatal dietary exposures on childhood development of atopic disease. 69. The effectiveness of maternal dietary interventions during pregnancy on obesity in offspring: A systematic review and meta-analysis. 70. The Importance of a Healthy Microbiome in Pregnancy and Infancy and Microbiota Treatment to Reverse Dysbiosis for Improved Health. 71. The maternal diet index in pregnancy is associated with offspring allergic diseases: the Healthy Start study. 72. The maternal environment and infant gut microbiome: A systematic review. 73. The pregnancy and birth to 24 months project: a series of systematic reviews on diet and health. 74. The Rationale for Probiotics Improving Reproductive Health and Pregnancy Outcome. 75. The Sporobiota of the Human Gut. 76. Therapeutic modalities for cow's milk allergy. 77. Understanding the role of probiotics and prebiotics in preventing allergic disease: evidence and methodological issues. 78. What's new in atopic eczema? An analysis of systematic reviews published in 2007 and 2008. Part 2. Disease prevention and treatment. 79. What's new in atopic eczema? An analysis of systematic reviews published in 2010-11. 80. What's new in atopic eczema? An analysis of systematic reviews published in 2018. Part 1: prevention and topical therapies. 81. What’s new in atopic eczema? An analysis of systematic reviews published in 2019. Part 1: Risk factors and prevention. |
| **Non-human study** |
| **Title** |
| 1. In utero Programming of Allergic Susceptibility. |
| **No relevant outcomes for offsprings** |
| **Title** |
| 1. Advancements in Nutritional Strategies for Gestational Diabetes Management: A Systematic Review of Recent Evidence. 2. Associations of dietary bioactive compounds with maternal adiposity and inflammation in gestational diabetes: An update on observational and clinical studies. 3. Breast milk microbiota: A review of the factors that influence composition. 4. Do probiotics in pregnancy reduce the risk of group B streptococcal colonisation? 5. Effects of Pre/Probiotic Supplementation on Breast Milk Levels of TGF-b1, TGF-b2, and IgA: A Systematic Review and Meta-Analysis of Randomized-Controlled Trial. 6. Efficacy of Direct or Indirect Use of Probiotics for the Improvement of Maternal Depression during Pregnancy and in the Postnatal Period: A Systematic Review and Meta-Analysis. 7. Interventions for preventing mastitis after childbirth. 8. Interventions to prevent women from developing gestational diabetes mellitus: an overview of Cochrane Reviews. 9. Maternal gut microbiota in the postpartum Period: A Systematic review. 10. Nutritional manipulation for the primary prevention of gestational diabetes mellitus: A meta-analysis of randomised studies. 11. Participant characteristics in the prevention of gestational diabetes as evidence for precision medicine: a systematic review and meta-analysis. 12. Probiotics in pregnancy and maternal outcomes: A systematic review. 13. The Effects of Probiotics/Synbiotics on Glucose and Lipid Metabolism in Women with Gestational Diabetes Mellitus: A Meta-Analysis of Randomized Controlled Trials. 14. The preventive and therapeutic effects of probiotics on mastitis: A systematic review and meta-analysis. |
| **The topics of the articles were not relevant to the review’s objective/theme** |
| **Title** |
| 1. A systematic review of quality and consistency of clinical practice guidelines on the primary prevention of food allergy and atopic dermatitis. 2. A systematic review of the effects of dietary interventions on neonatal outcomes in adolescent pregnancy. 3. Antenatal interventions for preventing stillbirth, fetal loss and perinatal death: an overview of Cochrane systematic reviews. 4. Complementary medicine products: Information sources, perceived benefits and maternal health literacy. 5. Dietary guidelines for pregnancy: A review of current evidence. 6. Do effects of early life interventions on linear growth correspond to effects on neurobehavioural development? A systematic review and meta-analysis. 7. Effect of women's nutrition before and during early pregnancy on maternal and infant outcomes: A systematic review. 8. Effectiveness of non-pharmacological interventions to prevent anemia in pregnant women: a quantitative systematic review protocol. 9. Effectiveness of Postnatal Maternal or Caregiver Interventions on Outcomes among Infants under Six Months with Growth Faltering: A Systematic Review. 10. Effects of Low Glycemic Index Diets on Gestational Diabetes Mellitus A Meta-Analysis of Randomized Controlled Clinical Trials. 11. Effects of nutrition interventions during pregnancy on low birth weight: an overview of systematic reviews. 12. Effects of nutritional interventions during pregnancy on birth, child health and development outcomes: A systematic review of evidence from low- and middle-income countries. 13. Effects of Nutritional Interventions during Pregnancy on Infant and Child Cognitive Outcomes: A Systematic Review and Meta-Analysis. 14. Enteral Bioactive Factor Supplementation in Preterm Infants: A Systematic Review. 15. Evidence for the Effects of Complementary Feeding Interventions on the Growth of Infants and Young Children in Low- and Middle-Income Countries. 16. Evidence-Based Updates on the First Week of Exclusive Breastfeeding Among Infants ≥35 Weeks. 17. Genetic and environmental risk factors for the development of food allergy. 18. How to Make a Young Child Smarter: Evidence From the Database of Raising Intelligence. 19. Maternal dietary diversity during pregnancy and risk of low birth weight in newborns: a systematic review. 20. Maternal lifestyle factors and risk of neuroblastoma in the offspring: A meta-analysis including Greek NARECHEM-ST primary data. 21. Maternal nutrient supplementation for suspected impaired fetal growth. 22. Maternal-focused interventions to improve infant growth and nutritional status in low-middle income countries: A systematic review of reviews. 23. Maternal, Infant, and Child Health Outcomes Associated With the Special Supplemental Nutrition Program for Women, Infants, and Children : A Systematic Review. 24. Mismatch between Probiotic Benefits in Trials versus Food Products. 25. Nutritional Gaps and Supplementation in the First 1000 Days. 26. Preconception and periconception interventions to prevent low birth weight, small for gestational age and preterm birth: a systematic review and meta-analysis. 27. Preconception Care. 28. Systematic Review of the Effect of Enteral Feeding on Gut Microbiota in Preterm Infants. 29. The effects of fermented food consumption in pregnancy on neonatal and infant health: An integrative review. 30. The Impact of Nutritional Interventions in Pregnant Women on DNA Methylation Patterns of the Offspring: A Systematic Review. 31. The neonatal intestinal resistome and factors that influence it-a systematic review. 32. Vitamin E supplementation in pregnancy. 33. Weight Gain and Nutrition during Pregnancy: An Analysis of Clinical Practice Guidelines in the Asia-Pacific Region. 34. Worldwide evidence about infant stunting from a public health perspective: A systematic review. |
| **Network meta-analysis** |
| **Title** |
| 1. Comparative effectiveness of probiotic strains on the prevention of pediatric atopic dermatitis: A systematic review and network meta-analysis. |
| **Administration of probiotics to the infants directly** |
| **Title** |
| 1. A systematic review of the safety of probiotics. 2. Association of Probiotics with Atopic Dermatitis among Infant: A Meta-analysis of Randomized Controlled Trials. 3. Comparison of common interventions for the treatment of infantile colic: a systematic review of reviews and guidelines. 4. Early probiotics to prevent childhood metabolic syndrome: A systematic review. 5. Effect of probiotic supplementation on cognitive function in children and adolescents: a systematic review of randomised trials. 6. Effects of maternal probiotic exposure during pregnancy and lactation on the mother and infant. 7. Evidence-Based Approaches to Minimize the Risk of Developing Necrotizing Enterocolitis in Premature Infants. 8. Impact of probiotic on anxiety and depression symptoms in pregnant and lactating women and microbiota of infants: A systematic review and meta-analysis. 9. Infant colic - What works: A systematic review of interventions for breast-fed infants. 10. Interventions to reduce preterm birth and stillbirth, and improve outcomes for babies born preterm in low- and middle-income countries: A systematic review. 11. Microbial effects of prebiotics, probiotics and synbiotics after Caesarean section or exposure to antibiotics in the first week of life: A systematic review. 12. Preventing food allergy in infancy and childhood: Systematic review of randomised controlled trials. 13. Probiotics for the Treatment of Infantile Colic: A Systematic Review. 14. Risk factors for necrotizing enterocolitis in neonates: A meta-analysis. 15. The impact of Caesarean section on the infant gut microbiome. |
| **Systematic reviews without suitable extractable data** |
| **Title** |
| 1. Effect of Probiotics on Metabolic Outcomes in Pregnant Women with Gestational Diabetes: A Systematic Review and Meta-Analysis of Randomized Controlled Trials. 2. Impact of maternal nutritional supplementation during pregnancy and lactation on the infant gut or breastmilk microbiota: A systematic review. 3. Maternal exposures and the infant gut microbiome: a systematic review with meta-analysis. 4. Supplementation of Probiotics in Pregnant Women Targeting Group B Streptococcus Colonization: A Systematic Review and Meta-Analysis. 5. The effect of probiotics on gestational diabetes and its complications in pregnant mother and newborn: A systematic review and meta-analysis during 2010-2020. 6. Can a probiotic supplement in pregnancy result in transfer to the neonatal gut: A systematic review. 7. Cohort profile update: the norwegian mother and child cohort study (MoBa). 8. Do probiotics effectively ameliorate glycemic control during gestational diabetes? A systematic review. 9. Do Probiotics in Pregnancy Reduce Allergies and Asthma in Infancy and Childhood? A Systematic Review. 10. Effect of nutrient supplementation on atopic dermatitis in children: a systematic review of probiotics, prebiotics, formula, and fatty acids. 11. Maternal Intake of Probiotics to Program Offspring Health. 12. Maternal Lifestyle Factors Affecting Breast Milk Composition and Infant Health: A Systematic Review. 13. Optimal nutrition in lactating women and its effect on later health of offspring: A systematic review of current evidence and recommendations (EarlyNutrition project). 14. Probiotic Supplementation during the Perinatal and Infant Period: Effects on gut Dysbiosis and Disease. 15. Systematic review of interventions in early pregnancy among pregnant individuals at risk for hyperglycemia. 16. The Impact of Probiotics, Prebiotics, and Synbiotics during Pregnancy or Lactation on the Intestinal Microbiota of Children Born by Cesarean Section: A Systematic Review. 17. The influence of probiotics on vaccine responses – A systematic review. 18. The Influences of Oral Probiotics on the Immunometabolic Response During Pregnancy and Lactation: A Systematic Review. |
| **Previous Cochrane reviews** |
| **Title** |
| 1. Maternal probiotic supplementation for prevention of morbidity and mortality in preterm infants. 2. Probiotic treatment for women with gestational diabetes to improve maternal and infant health and well-being. 3. Probiotics for preventing gestational diabetes. |
| **Had overlapping meta-analyses on maternal probiotic supplementation and the same outcomes** |
| **Title** |
| 1. Are probiotics and prebiotics safe for use during pregnancy and lactation? A systematic review and meta-analysis. 2. Can mixed strains of Lactobacillus and Bifidobacterium reduce eczema in infants under three years of age? A meta-analysis. 3. Diet and pre-intervention washout modifies the effects of probiotics on gestational diabetes mellitus: A comprehensive systematic review and meta-analysis of randomized controlled trials. 4. Diet during pregnancy and infancy and risk of allergic or autoimmune disease: A systematic review and meta-analysis. 5. Effects of Probiotic Supplement in Pregnant Women with Gestational Diabetes Mellitus: A Systematic Review and Meta-Analysis of Randomized Controlled Trials. 6. Meta-analysis of probiotics and/or prebiotics for the prevention of eczema. 7. Meta-analysis on preventive and therapeutic effects of probiotic supplementation in infant atopic dermatitis. 8. Probiotic administration in early life, atopy, and asthma: a meta-analysis of clinical trials. 9. Probiotics and primary prevention of atopic dermatitis: a meta-analysis of randomized controlled studies. 10. Probiotics for preventing gestational diabetes mellitus in overweight or obese pregnant women: A systematic review and meta-analysis. 11. Probiotics for the prevention of atopic dermatitis in infants from different geographic regions: a systematic review and Meta-analysis. 12. Probiotics supplementation during pregnancy or infancy for the prevention of atopic dermatitis: A meta-analysis. 13. Probiotics supplementation during pregnancy or infancy on multiple food allergies and gut microbiota: a systematic review and meta-analysis. 14. Systematic review and meta-analysis on the use of probiotic supplementation in pregnant mother, breastfeeding mother and infant for the prevention of atopic dermatitis in children. 15. The Role of Probiotics in the Prevention and Treatment of Atopic Dermatitis in Children: An Updated Systematic Review and Meta-Analysis of Randomized Controlled Trials. |
| **Meta-analyses not included in quantitative synthesis** |
| **Title** |
| 1. Lactobacillus rhamnosus GG in the Primary Prevention of Eczema in Children: A Systematic Review and Meta-Analysis. 2. Probiotic supplementation during pregnancy or infancy for the prevention of asthma and wheeze: systematic review and meta-analysis. 3. Probiotic Supplementation for Prevention of Atopic Dermatitis in Infants and Children: A Systematic Review and Meta-analysis. 4. Probiotics for Prevention of Atopy and Food Hypersensitivity in Early Childhood: A PRISMA-Compliant Systematic Review and Meta-Analysis of Randomized Controlled Trials. 5. Probiotics for the prevention of allergy: A systematic review and meta-analysis of randomized controlled trials. |

**GRADE ratings of associations between maternal probiotic supplementation and offspring outcomes**

**Pregnancy**

**Author(s):** SWR

**Question:** Probiotics compared to control for women during pregnancy

| **Certainty assessment** | | | | | | | **№ of patients** | | **Effect** | | **Certainty** | **Importance** |
| --- | --- | --- | --- | --- | --- | --- | --- | --- | --- | --- | --- | --- |
| **№ of studies** | **Study design** | **Risk of bias** | **Inconsistency** | **Indirectness** | **Imprecision** | **Other considerations** | **probiotics** | **control** | **Relative (95% CI)** | **Absolute (95% CI)** |  |  |
| **Birth weight-Saeed Baradwan, 2023（4） (assessed with: SMD)** | | | | | | | | | | | | |
| 4 | randomised trials | serious | not serious | not serious | serious^a^ | none | 166 | 173 | - | SMD **0.36 SD more** (0.12 more to 0.61 more) | ⨁⨁◯◯ Low^a^ |  |
| **Birth weight-Alexander Jarde，2018（4） (assessed with: SMD)** | | | | | | | | | | | | |
| 10 | randomised trials | not serious | not serious | not serious | serious^b^ | publication bias strongly suspected^c^ | 840 | 768 | - | SMD **0.03 SD more** (0.07 fewer to 0.13 more) | ⨁⨁◯◯ Low^b,c^ |  |
| **Birth weight-Karaponi Am Okesene-Gafa，2020（8） (assessed with: SMD)** | | | | | | | | | | | | |
| 4 | randomised trials | not serious | not serious | not serious | serious^a^ | none | 161 | 163 | - | SMD **0.15 SD fewer** (0.37 fewer to 0.06 more) | ⨁⨁⨁◯ Moderate^a^ |  |
| **Birth weight-Bekalu Kassie Alemu，2023（5） (assessed with: SMD)** | | | | | | | | | | | | |
| 2 | randomised trials | not serious | not serious | not serious | very serious^a,d^ | none | 74 | 71 | - | SMD **0.5 SD more** (0.83 fewer to 0.17 fewer) | ⨁⨁◯◯ Low^a,d^ |  |
| **Birth weight-Chun-Chi Wang，2020 (assessed with: MD)** | | | | | | | | | | | | |
| 3 | randomised trials | not serious | not serious | not serious | very serious^a,b^ | none |  |  | - | MD **50.6 grams more** (68.04 fewer to 169.25 more) | ⨁⨁◯◯ Low^a,b^ |  |
| **Caesarean-Karaponi Am Okesene-Gafa，2020（1） (assessed with: RR)** | | | | | | | | | | | | |
| 3 | randomised trials | not serious | not serious | not serious | very serious^a,e^ | none | 35/133 (26.3%) | 47/134 (35.1%) | **RR 0.64** (0.30 to 1.35) | **126 fewer per 1,000** (from 246 fewer to 123 more) | ⨁⨁◯◯ Low^a,e^ |  |
| **Caesarean-Sarah J Davidson，2021（2） (assessed with: RR)** | | | | | | | | | | | | |
| 2 | randomised trials | not serious | not serious | not serious | serious^b,e^ | none | 93/269 (34.6%) | 105/278 (37.8%) | **RR 0.91** (0.73 to 1.14) | **34 fewer per 1,000** (from 102 fewer to 53 more) | ⨁⨁⨁◯ Moderate^b,e^ |  |
| **Caesarean-Alexander Jarde，2018（35） (assessed with: RR)** | | | | | | | | | | | | |
| 9 | randomised trials | not serious | not serious | not serious | serious^b^ | none | 127/639 (19.9%) | 147/569 (25.8%) | **RR 0.83** (0.67 to 1.04) | **44 fewer per 1,000** (from 85 fewer to 10 more) | ⨁⨁⨁◯ Moderate^b^ |  |
| **Detection rate of beneficial bacteria in breast milk by mothers using only during pregnancy-Bekalu Kassie Alemu，2023（9） (assessed with: RR)** | | | | | | | | | | | | |
| 6 | randomised trials | not serious | serious^f^ | not serious | serious^a^ | none | 146/328 (44.5%) | 107/326 (32.8%) | **RR 1.95** (1.03 to 3.66) | **312 more per 1,000** (from 10 more to 873 more) | ⨁⨁◯◯ Low^a,f^ |  |
| **Gestational age at birth-Saeed Baradwan, 2023（1） (assessed with: SMD)** | | | | | | | | | | | | |
| 4 | randomised trials | serious^g^ | not serious | not serious | serious^a,d^ | none | 166 | 173 | - | SMD **0.63 SD more** (0.03 more to 1.23 more) | ⨁⨁◯◯ Low^a,d,g^ |  |
| **Gestational age at birth-Jacquelyn Grev，2018（8） (assessed with: SMD)** | | | | | | | | | | | | |
| 2 | randomised trials | not serious | not serious | not serious | serious^b,d^ | none | 103 | 104 | - | SMD **0.08 SD more** (0.19 fewer to 0.36 more) | ⨁⨁⨁◯ Moderate^b,d^ |  |
| **Gestational age at birth-Alexander Jarde，2018（3） (assessed with: SMD)** | | | | | | | | | | | | |
| 8 | randomised trials | not serious | not serious | not serious | serious^b^ | none | 599 | 534 | - | SMD **0.05 SD more** (0.07 fewer to 0.16 more) | ⨁⨁⨁◯ Moderate^b^ |  |
| **Head circumference-Karaponi Am Okesene-Gafa，2020（9） (assessed with: SMD)** | | | | | | | | | | | | |
| 3 | randomised trials | not serious | not serious | not serious | serious^b,d^ | none | 124 | 125 | - | SMD **0 SD**  (0.25 lower to 0.25 higher) | ⨁⨁⨁◯ Moderate^b,d^ |  |
| **Hyperbilirubinemia-Karaponi Am Okesene-Gafa，2020（12） (assessed with: RR)** | | | | | | | | | | | | |
| 2 | randomised trials | not serious | not serious | not serious | extremely serious^a,h^ | none | 3/60 (5.0%) | 17/60 (28.3%) | **RR 0.19** (0.06 to 0.61) | **230 fewer per 1,000** (from 266 fewer to 111 fewer) | ⨁◯◯◯ Very low^a,h^ |  |
| **Hypoglycemia-Karaponi Am Okesene-Gafa，2020（11） (assessed with: RR)** | | | | | | | | | | | | |
| 3 | randomised trials | not serious | not serious | not serious | extremely serious^a,b,h^ | none | 10/88 (11.4%) | 12/89 (13.5%) | **RR 0.84** (0.38 to 1.83) | **22 fewer per 1,000** (from 84 fewer to 112 more) | ⨁◯◯◯ Very low^a,b,h^ |  |
| **Induction of labor-Sarah J Davidson，2021（18） (assessed with: RR)** | | | | | | | | | | | | |
| 2 | randomised trials | not serious | not serious | not serious | serious^b,e^ | none | 89/268 (33.2%) | 84/276 (30.4%) | **RR 1.06** (0.78 to 1.46) | **18 more per 1,000** (from 67 fewer to 140 more) | ⨁⨁⨁◯ Moderate^b,e^ |  |
| **Latency period duration-Saeed Baradwan, 2023（2） (assessed with: SMD)** | | | | | | | | | | | | |
| 3 | randomised trials | serious^g^ | not serious | not serious | serious^a,b^ | none | 136 | 143 | - | SMD **0.72 SD more** (0.02 fewer to 1.46 more) | ⨁⨁◯◯ Low^a,b,g^ |  |
| **LGA-Karaponi Am Okesene-Gafa，2020（2） (assessed with: RR)** | | | | | | | | | | | | |
| 2 | randomised trials | not serious | not serious | not serious | extremely serious^a,b,h^ | none | 10/86 (11.6%) | 14/88 (15.9%) | **RR 0.71** (0.34 to 1.51) | **46 fewer per 1,000** (from 105 fewer to 81 more) | ⨁◯◯◯ Very low^a,b,h^ |  |
| **LGA-Sarah J Davidson，2021（10） (assessed with: RR)** | | | | | | | | | | | | |
| 2 | randomised trials | not serious | not serious | not serious | very serious^b,e^ | none | 41/255 (16.1%) | 37/254 (14.6%) | **RR 1.08** (0.72 to 1.62) | **12 more per 1,000** (from 41 fewer to 90 more) | ⨁⨁◯◯ Low^b,e^ |  |
| **Macrosomia-Karaponi Am Okesene-Gafa，2020（6） (assessed with: RR)** | | | | | | | | | | | | |
| 3 | randomised trials | not serious | not serious | not serious | extremely serious^a,b,h^ | none | 19/133 (14.3%) | 23/134 (17.2%) | **RR 0.50** (0.11 to 2.35) | **86 fewer per 1,000** (from 153 fewer to 232 more) | ⨁◯◯◯ Very low^a,b,h^ |  |
| **Misscharge/stillbirth-Jacquelyn Grev，2018（10） (assessed with: RR)** | | | | | | | | | | | | |
| 2 | randomised trials | not serious | not serious | not serious | extremely serious^a,b,h^ | none | 3/159 (1.9%) | 4/161 (2.5%) | **RR 0.82** (0.13 to 5.05) | **4 fewer per 1,000** (from 22 fewer to 101 more) | ⨁◯◯◯ Very low^a,b,h^ |  |
| **Mortality and morbidity-Sarah J Davidson，2021（17） (assessed with: RR)** | | | | | | | | | | | | |
| 2 | randomised trials | not serious | not serious | not serious | extremely serious^a,b,h^ | none | 13/313 (4.2%) | 19/310 (6.1%) | **RR 0.69** (0.36 to 1.34) | **19 fewer per 1,000** (from 39 fewer to 21 more) | ⨁◯◯◯ Very low^a,b,h^ |  |
| **Neonatal mortality-Jacquelyn Grev，2018（9） (assessed with: RD)** | | | | | | | | | | | | |
| 2 | randomised trials | not serious | not serious | not serious | extremely serious^a,b,h^ | none | 0/144 (0.0%) | 0/154 (0.0%) | **RD 0.00** (-0.02 to 0.02) | **-- per 1,000** (from -- to --) | ⨁◯◯◯ Very low^a,b,h^ |  |
| **Neonatal length-Alexander Jarde，2018（31） (assessed with: SMD)** | | | | | | | | | | | | |
| 4 | randomised trials | not serious | not serious | not serious | serious^a,b^ | none | 262 | 266 | - | SMD **0.08 SD more** (0.13 fewer to 0.29 more) | ⨁⨁⨁◯ Moderate^a,b^ |  |
| **Neonatal sepsis-Saeed Baradwan, 2023（6） (assessed with: RR)** | | | | | | | | | | | | |
| 3 | randomised trials | serious^g^ | not serious | not serious | extremely serious^b,h^ | none | 20/133 (15.0%) | 27/139 (19.4%) | **RR 0.75** (0.45 to 1.27) | **49 fewer per 1,000** (from 107 fewer to 52 more) | ⨁◯◯◯ Very low^b,g,h^ |  |
| **NICU-Alexander Jarde，2018（34） (assessed with: RR)** | | | | | | | | | | | | |
| 3 | randomised trials | not serious | not serious | not serious | extremely serious^b,h^ | none | 24/182 (13.2%) | 25/195 (12.8%) | **RR 1.03** (0.62 to 1.72) | **4 more per 1,000** (from 49 fewer to 92 more) | ⨁◯◯◯ Very low^b,h^ |  |
| **Polyhydramnios-Rui Wu，2024（11） (assessed with: RR)** | | | | | | | | | | | | |
| 3 | randomised trials | not serious | not serious | not serious | extremely serious^a,b,h^ | none | 3/89 (3.4%) | 7/88 (8.0%) | **RR 0.46** (0.14 to 1.57) | **43 fewer per 1,000** (from 68 fewer to 45 more) | ⨁◯◯◯ Very low^a,b,h^ |  |
| **PPROM-Alexander Jarde，2018（7） (assessed with: RR)** | | | | | | | | | | | | |
| 2 | randomised trials | not serious | not serious | not serious | extremely serious^a,b,h^ | none | 14/183 (7.7%) | 10/183 (5.5%) | **RR 1.37** (0.63 to 2.99) | **20 more per 1,000** (from 20 fewer to 109 more) | ⨁◯◯◯ Very low^a,b,h^ |  |
| **Preterm birth < 34 weeks-Annie McDougall，2024 (assessed with: RR)** | | | | | | | | | | | | |
| 3 | randomised trials | not serious | not serious | not serious | extremely serious^a,b,h^ | none | 7/522 (1.3%) | 6/505 (1.2%) | **RR 1.15** (0.37 to 3.52) | **2 more per 1,000** (from 7 fewer to 30 more) | ⨁◯◯◯ Very low^a,b,h^ |  |
| **Preterm birth < 34 weeks-Jacquelyn Grev，2018（7） (assessed with: RD)** | | | | | | | | | | | | |
| 2 | randomised trials | not serious | not serious | not serious | extremely serious^a,b,h^ | none | 0/137 (0.0%) | 0/150 (0.0%) | **RD 0.00** (-0.02 to 0.02) | **-- per 1,000** (from -- to --) | ⨁◯◯◯ Very low^a,b,h^ |  |
| **Preterm birth < 37 weeks-Alexander Jarde，2018（2） (assessed with: RR)** | | | | | | | | | | | | |
| 11 | randomised trials | not serious | not serious | not serious | very serious^b,e^ | none | 58/1250 (4.6%) | 42/1234 (3.4%) | **RR 1.08** (0.71 to 1.63) | **3 more per 1,000** (from 10 fewer to 21 more) | ⨁⨁◯◯ Low^b,e^ |  |
| **RDS-Saeed Baradwan, 2023（7） (assessed with: RR)** | | | | | | | | | | | | |
| 3 | randomised trials | serious^g^ | not serious | not serious | extremely serious^b,h^ | none | 28/133 (21.1%) | 38/139 (27.3%) | **RR 0.75** (0.50 to 1.13) | **68 fewer per 1,000** (from 137 fewer to 36 more) | ⨁◯◯◯ Very low^b,g,h^ |  |
| **SGA-Alexander Jarde，2018（5） (assessed with: RR)** | | | | | | | | | | | | |
| 3 | randomised trials | not serious | not serious | not serious | extremely serious^a,b,h^ | none | 17/152 (11.2%) | 18/166 (10.8%) | **RR 1.02** (0.35 to 2.99) | **2 more per 1,000** (from 70 fewer to 216 more) | ⨁◯◯◯ Very low^a,b,h^ |  |

**CI:** confidence interval; **MD:** mean difference; **RR:** risk ratio; **SMD:** standardised mean difference

#### Explanations

a. Too wide 95% confidence interval

b. The 95% confidence interval includes invalid value

c. P value of Egger's test is＜0.1

d. The sample size is too small

e. The number of events is too small

f. The authors do not fully explain plausible reasons for the high heterogeneity

g. All studies have high risk performance bias and detection bias

h. The number of events is extremely small

**Lactation**

**Author(s):** SWR

**Question:** Probiotics compared to control for women during lactation

| **Certainty assessment** | | | | | | | **№ of patients** | | **Effect** | | **Certainty** | **Importance** |
| --- | --- | --- | --- | --- | --- | --- | --- | --- | --- | --- | --- | --- |
| **№ of studies** | **Study design** | **Risk of bias** | **Inconsistency** | **Indirectness** | **Imprecision** | **Other considerations** | **probiotics** | **control** | **Relative (95% CI)** | **Absolute (95% CI)** |  |  |
| **Detection rate of beneficial bacteria in breast milk by lactating mothers with illness-Bekalu Kassie Alemu，2023（8） (assessed with: RR)** | | | | | | | | | | | | |
| 4 | randomised trials | not serious | serious^a^ | not serious | very serious^b,c,d^ | none | 174/343 (50.7%) | 45/267 (16.9%) | RR 2.34 (0.93 to 5.87) | **226 more per 1,000** (from 12 fewer to 821 more) | ⨁◯◯◯ Very low^a,b,c,d^ |  |
| **Detection rate of beneficial bacteria in breast milk by mothers using only during lactation-Bekalu Kassie Alemu，2023（10） (assessed with: RR)** | | | | | | | | | | | | |
| 6 | randomised trials | not serious | serious^a^ | not serious | serious^c^ | none | 194/371 (52.3%) | 47/284 (16.5%) | RR 2.70 (1.22 to 5.96) | **281 more per 1,000** (from 36 more to 821 more) | ⨁⨁◯◯ Low^a,c^ |  |

**CI:** confidence interval; **RR:** risk ratio

#### Explanations

a. The authors do not fully explain plausible reasons for the high heterogeneity

b. The 95% confidence interval includes invalid value

c. The 95% confidence interval is too wide

d. The number of events is too small

**Pregnancy and lactation**

**Author(s):**

**Question:** Probiotics compared to control for women from pregnancy to lactation

| **Certainty assessment** | | | | | | | **№ of patients** | | **Effect** | | **Certainty** | **Importance** |
| --- | --- | --- | --- | --- | --- | --- | --- | --- | --- | --- | --- | --- |
| **№ of studies** | **Study design** | **Risk of bias** | **Inconsistency** | **Indirectness** | **Imprecision** | **Other considerations** | **probiotics** | **control** | **Relative (95% CI)** | **Absolute (95% CI)** |  |  |
| **Infant stool beneficial bacteria abundance-Bekalu Kassie Alemu，2023（4） (assessed with: SMD)** | | | | | | | | | | | | |
| 6 | randomised trials | not serious | serious^a^ | not serious | serious^b^ | none | 485 | 409 | - | SMD **0.89 SD more** (0.38 more to 1.4 more) | ⨁⨁◯◯ Low^a,b^ |  |
| **Colic-Bekalu Kassie Alemu，2023（6） (assessed with: RR)** | | | | | | | | | | | | |
| 3 | randomised trials | not serious | not serious | not serious | very serious^b,c^ | none | 10/187 (5.3%) | 35/201 (17.4%) | **RR 0.30** (0.16 to 0.57) | **122 fewer per 1,000** (from 146 fewer to 75 fewer) | ⨁⨁◯◯ Low^b,c^ |  |
| **Breastfeeding at 6 months-Sarah J Davidson，2021（19） (assessed with: RR)** | | | | | | | | | | | | |
| 2 | randomised trials | not serious | not serious | not serious | serious^d^ | none | 221/279 (79.2%) | 220/273 (80.6%) | **RR 0.99** (0.91 to 1.07) | **8 fewer per 1,000** (from 73 fewer to 56 more) | ⨁⨁⨁◯ Moderate^d^ |  |
| **Detection rate of beneficial bacteria in breastmilk-Bekalu Kassie Alemu，2023（1） (assessed with: RR)** | | | | | | | | | | | | |
| 12 | randomised trials | not serious | serious^a^ | not serious | not serious | publication bias strongly suspected | 340/699 (48.6%) | 154/609 (25.3%) | **RR 1.80** (1.25 to 2.58) | **202 more per 1,000** (from 63 more to 400 more) | ⨁⨁◯◯ Low^a^ |  |
| **The mean abundance of beneficial bacteria in breast milk（log10 CFU/mL for beneficial bacteria）-Bekalu Kassie Alemu，2023（2） (assessed with: SMD)** | | | | | | | | | | | | |
| 10 | randomised trials | not serious | serious^a^ | not serious | serious^b^ | publication bias strongly suspected | 433 | 436 | - | SMD **1.22 SD more** (0.62 more to 1.83 more) | ⨁◯◯◯ Very low^a,b^ |  |
| **The mean abundance of pathogenic bacteria in breast milk（log10 CFU/mL for pathogenic bacteria）-Bekalu Kassie Alemu，2023（3） (assessed with: SMD)** | | | | | | | | | | | | |
| 6 | randomised trials | not serious | not serious | not serious | serious^b^ | none | 385 | 360 | - | SMD **0.9 SD fewer** (1.49 fewer to 0.31 fewer) | ⨁⨁⨁◯ Moderate^b^ |  |
| **Atopic dermatitis-Jeffrey Voigt，2022（5） (assessed with: RR)** | | | | | | | | | | | | |
| 6 | randomised trials | not serious | not serious | not serious | not serious | none | 97/598 (16.2%) | 169/582 (29.0%) | **RR 0.52** (0.38 to 0.72) | **139 fewer per 1,000** (from 180 fewer to 81 fewer) | ⨁⨁⨁⨁ High |  |
| **Eczema-G Zuccotti，2015（2） (assessed with: RR)** | | | | | | | | | | | | |
| 3 | randomised trials | not serious | not serious | not serious | serious^d^ | none | 128/437 (29.3%) | 142/431 (32.9%) | **RR 0.89** (0.73 to 1.08) | **36 fewer per 1,000** (from 89 fewer to 26 more) | ⨁⨁⨁◯ Moderate^d^ |  |
| **Eczema-G Zuccotti，2015（3） (assessed with: RR)** | | | | | | | | | | | | |
| 10 | randomised trials | not serious | not serious | not serious | not serious | none | 213/789 (27.0%) | 282/778 (36.2%) | **RR 0.75** (0.57 to 0.97) | **91 fewer per 1,000** (from 156 fewer to 11 fewer) | ⨁⨁⨁⨁ High |  |
| **Rhinoconjunctivitis-G Zuccotti，2015（4） (assessed with: RR)** | | | | | | | | | | | | |
| 5 | randomised trials | not serious | not serious | not serious | serious^d^ | none | 106/790 (13.4%) | 78/511 (15.3%) | **RR 0.90** (0.64 to 1.27) | **15 fewer per 1,000** (from 55 fewer to 41 more) | ⨁⨁⨁◯ Moderate^d^ |  |

**CI:** confidence interval; **RR:** risk ratio; **SMD:** standardised mean difference

#### Explanations

a. The authors do not fully explain plausible reasons for the high heterogeneity

b. The 95% confidence interval is too wide

c. The number of events is extremely small

d. The 95% confidence interval includes invalid value

**Pregnancy and/or lactation**

**Author(s):** SWR

**Question:** Probiotics compared to control for women during pregnancy and/or lactation

| **Certainty assessment** | | | | | | | **№ of patients** | | **Effect** | | **Certainty** | **Importance** |
| --- | --- | --- | --- | --- | --- | --- | --- | --- | --- | --- | --- | --- |
| **№ of studies** | **Study design** | **Risk of bias** | **Inconsistency** | **Indirectness** | **Imprecision** | **Other considerations** | **probiotics** | **control** | **Relative (95% CI)** | **Absolute (95% CI)** |  |  |
| **Atopic eczema-Shuya Sun，2022（4） (assessed with: RR)** | | | | | | | | | | | | |
| 3 | randomised trials | not serious | not serious | not serious | very serious^a^ | none | 55/317 (17.4%) | 79/309 (25.6%) | **RR 0.68** (0.50 to 0.93) | **82 fewer per 1,000** (from 128 fewer to 18 fewer) | ⨁⨁◯◯ Low^a^ |  |
| **Atopic dermatitis-Feina Wang，2023（2） (assessed with: RR)** | | | | | | | | | | | | |
| 7 | randomised trials | not serious | serious^b^ | not serious | serious^c^ | none |  |  | **RR 0.58** (0.25 to 0.90) | **1 fewer per 1,000** (from 1 fewer to 0 fewer) | ⨁⨁◯◯ Low^b,c^ |  |
| **Birthweight-Sarah J Davidson，2021（26） (assessed with: SMD)** | | | | | | | | | | | | |
| 6 | randomised trials | not serious | not serious | not serious | serious^d^ | none | 761 | 763 | - | SMD **0.05 SD more** (0.08 fewer to 0.18 more) | ⨁⨁⨁◯ Moderate^d^ |  |
| **Caesarean-Sarah J Davidson，2021（1） (assessed with: RR)** | | | | | | | | | | | | |
| 6 | randomised trials | not serious | not serious | not serious | serious^d^ | none | 216/758 (28.5%) | 217/762 (28.5%) | **RR 1.00** (0.86 to 1.17) | **0 fewer per 1,000** (from 40 fewer to 48 more) | ⨁⨁⨁◯ Moderate^d^ |  |
| **Eczema-Shuya Sun，2022（2） (assessed with: RR)** | | | | | | | | | | | | |
| 5 | randomised trials | not serious | not serious | not serious | not serious | none | 143/219 (65.3%) | 219/506 (43.3%) | **RR 0.61** (0.44 to 0.85) | **169 fewer per 1,000** (from 242 fewer to 65 fewer) | ⨁⨁⨁⨁ High |  |
| **Gestational at birth-Sarah J Davidson，2021（22） (assessed with: SMD)** | | | | | | | | | | | | |
| 5 | randomised trials | not serious | not serious | not serious | serious^d^ | none | 536 | 537 | - | SMD **0.01 SD more** (0.13 fewer to 0.15 more) | ⨁⨁⨁◯ Moderate^d^ |  |
| **Head circumference-Sarah J Davidson，2021（27） (assessed with: SMD)** | | | | | | | | | | | | |
| 3 | randomised trials | not serious | not serious | not serious | serious^d^ | none | 396 | 393 | - | SMD **0.02 SD fewer** (0.12 fewer to 0.08 more) | ⨁⨁⨁◯ Moderate^d^ |  |
| **Hyperbilirubinaemia-Sarah J Davidson，2021（33） (assessed with: RR)** | | | | | | | | | | | | |
| 2 | randomised trials | not serious | not serious | not serious | very serious^a,d^ | none | 47/301 (15.6%) | 48/292 (16.4%) | **RR 0.96** (0.64 to 1.44) | **7 fewer per 1,000** (from 59 fewer to 72 more) | ⨁⨁◯◯ Low^a,d^ |  |
| **Hypoglycemia-Sarah J Davidson，2021（32） (assessed with: RR)** | | | | | | | | | | | | |
| 2 | randomised trials | not serious | not serious | not serious | extremely serious^a,c,d^ | none | 45/297 (15.2%) | 39/289 (13.5%) | **RR 1.15** (0.69 to 1.92) | **20 more per 1,000** (from 42 fewer to 124 more) | ⨁◯◯◯ Very low^a,c,d^ |  |
| **Length-Sarah J Davidson，2021（28） (assessed with: SMD)** | | | | | | | | | | | | |
| 3 | randomised trials | not serious | serious^b^ | not serious | serious^d^ | none | 396 | 390 | - | SMD **0.01 SD higher** (0.22 lower to 0.23 higher) | ⨁⨁◯◯ Low^b,d^ |  |
| **LGA-Sarah J Davidson，2021（9） (assessed with: RR)** | | | | | | | | | | | | |
| 4 | randomised trials | not serious | not serious | not serious | very serious^a,d^ | none | 66/461 (14.3%) | 65/458 (14.2%) | **RR 1.00** (0.73 to 1.37) | **0 fewer per 1,000** (from 38 fewer to 53 more) | ⨁⨁◯◯ Low^a,d^ |  |
| **Macrosomia-Sarah J Davidson，2021（24） (assessed with: RR)** | | | | | | | | | | | | |
| 3 | randomised trials | not serious | not serious | not serious | very serious^a,d^ | none | 92/473 (19.5%) | 83/479 (17.3%) | **RR 1.13** (0.83 to 1.53) | **23 more per 1,000** (from 29 fewer to 92 more) | ⨁⨁◯◯ Low^a,d^ |  |
| **NICU-Sarah J Davidson，2021（43） (assessed with: RR)** | | | | | | | | | | | | |
| 5 | randomised trials | not serious | not serious | not serious | very serious^a,d^ | none | 95/677 (14.0%) | 97/677 (14.3%) | **RR 0.97** (0.75 to 1.26) | **4 fewer per 1,000** (from 36 fewer to 37 more) | ⨁⨁◯◯ Low^a,d^ |  |
| **Perinatal mortality (stillbirth and neonatal mortality)-Sarah J Davidson，2021（16） (assessed with: RR)** | | | | | | | | | | | | |
| 3 | randomised trials | not serious | not serious | not serious | extremely serious^c,d,e^ | none | 0/351 (0.0%) | 1/358 (0.3%) | **RR 0.33** (0.01 to 8.02) | **2 fewer per 1,000** (from 3 fewer to 20 more) | ⨁◯◯◯ Very low^c,d,e^ |  |
| **Ponderal index (kg/m3)-Sarah J Davidson，2021（29） (assessed with: SMD)** | | | | | | | | | | | | |
| 2 | randomised trials | not serious | not serious | not serious | serious^d^ | none | 266 | 273 | - | SMD **0.1 SD more** (0.07 fewer to 0.26 more) | ⨁⨁⨁◯ Moderate^d^ |  |
| **Preterm birth-Sarah J Davidson，2021（23） (assessed with: RR)** | | | | | | | | | | | | |
| 6 | randomised trials | not serious | not serious | not serious | very serious^a,d^ | none | 47/746 (6.3%) | 35/738 (4.7%) | **RR 1.32** (0.85 to 2.03) | **15 more per 1,000** (from 7 fewer to 49 more) | ⨁⨁◯◯ Low^a,d^ |  |
| **Preterm birth＜37 weeks' gestation-Jacquelyn Grev，2018（1） (assessed with: RR)** | | | | | | | | | | | | |
| 4 | randomised trials | not serious | not serious | not serious | extremely serious^c,d,e^ | none | 6/252 (2.4%) | 7/266 (2.6%) | **RR 1.02** (0.29 to 3.63) | **1 more per 1,000** (from 19 fewer to 69 more) | ⨁◯◯◯ Very low^c,d,e^ |  |
| **SGA-Sarah J Davidson，2021（25） (assessed with: RR)** | | | | | | | | | | | | |
| 3 | randomised trials | not serious | not serious | not serious | very serious^e^ | none | 20/411 (4.9%) | 39/403 (9.7%) | **RR 0.51** (0.30 to 0.87) | **47 fewer per 1,000** (from 68 fewer to 13 fewer) | ⨁⨁◯◯ Low^e^ |  |

**CI:** confidence interval; **RR:** risk ratio; **SMD:** standardised mean difference

#### Explanations

a. The number of events is too small.

b. The authors do not fully explain plausible reasons for the high heterogeneity

c. The 95% confidence interval is too wide.

d. The 95% confidence interval includes invalid value.

e. The number of events is extremely small.
